# Integrated Analysis of the Alterations in Gut Microbiota and Metabolites of Mice Induced After Long-Term Intervention With Different Antibiotics

**DOI:** 10.3389/fmicb.2022.832915

**Published:** 2022-06-29

**Authors:** Nan Zhang, Jun Liu, Zhiyun Chen, Ning Chen, Fangyan Gu, Qiushui He

**Affiliations:** ^1^Department of Medical Microbiology, Capital Medical University, Beijing, China; ^2^Laboratory Animal Center of Chinese PLA General Hospital, Beijing, China; ^3^Clinical Biobank Center of Chinese PLA General Hospital, Beijing, China; ^4^Institute of Biomedicine, University of Turku, Turku, Finland; ^5^InFLAMES Research Flagship Center, University of Turku, Turku, Finland

**Keywords:** microbial metabolomics, gut microbiota, inflammatory response, antibiotics, vancomycin, polymyxin B, mice

## Abstract

**Objectives:**

We aimed to study the effect of antibiotic-induced disruption of gut microbiome on host metabolomes and inflammatory responses after long-term use of antibiotics.

**Methods:**

A total of three groups of 3-week-old female C57BL/6 mice (*n* = 44) were continuously treated with vancomycin (VAN), polymyxin B (PMB), or water, respectively, for up to 28 weeks. Fecal samples collected at different time points were analyzed by bacterial 16S rRNA gene sequencing and untargeted metabolomics by ultraperformance liquid chromatography coupled with quadrupole time-of-flight tandem mass spectrometry (UPLC Q-TOF MS). Serum cytokines (IFN-γ, IL-2, IL-10, IL-13, IL-17A, and TNF-α) were determined by multiplex immunoassay.

**Results:**

Treatment by VAN or PMB did not affect the average body weight of mice. However, a heavier caecum observed in VAN-treated mice. Compared with PMB-treated and control mice, VAN treatment induced more rapid dysbiosis of gut microbiota and dysmetabolism. Instead of *Bacteroides*, VAN-treated mice had a compositional shift to *Proteobacteria* and its species *Escherichia coli* and *Verrucomicrobia* and its species *Akkermansia muciniphila*. The shift was accompanied by decreased richness and diversity in microbiota. PMB-treated mice had an increased *Firmicutes*, and the diversity was shortly increased and further decreased to the baseline. Decreased levels of short-chain and long-chain fatty acids, bile acids, L-arginine, dopamine, L-tyrosine, and phosphatidylcholine (all *p* < 0.05) were observed in VAN-treated mice. In contrast, significantly increased levels of amino acids including L-aspartic acid, beta-alanine, 5-hydroxy-L-tryptophan, L-glutamic acid, and lysophosphatidylcholines (all *p* < 0.05) were found. These changes occurred after 3-week treatment and remained unchanged up to 28 weeks. For PMB-treated mice, metabolites involved in the metabolic pathway of vitamin B6 were decreased, whereas glycocholic acid and chenodeoxycholic acid were increased (all *p* < 0.05). After 8-week treatment, VAN-treated mice had significantly higher levels of serum IFN-γ, IL-13, and IL-17A, and PMB-treated mice had higher levels of IL-13 and IL-17 compared to control mice. At 28-week treatment, only IL-17A remained high in PMB-treated mice.

**Conclusion:**

This study showed that the antibiotic-induced alterations in gut microbiota contribute to host inflammatory responses through the change in metabolic status, which are likely related to the type, rather than timing of antibiotic used.

## Introduction

An increasing number of evidence indicated that antibiotic-induced dysbiosis of gut microbiota is related to the dysfunctional metabolism in gut and blood. We have previously shown that female mice treated by vancomycin for 3 weeks had a reduction of *Bacteroidetes* and *Firmicutes* and an increase in *Proteobacteria* with *Enterobacteriaceae* family and *Verrucomicrobia* with *Akkermansiaceae* family. Moreover, changes in the abundance of these bacteria are correlated with the decreased fecal long-chain fatty acids (LCFAs) (Sun et al., 2019). However, there was no significant difference in gut microbiota and metabolites observed after the short-term treatment by polymyxin B (PMB) in the study. Zarrinpar et al. reported that use of antibiotic mixture (ampicillin, vancomycin, neomycin, metronidazole, and amphotericin B) in mice decreased the relative abundance of *Firmicutes* and *Bacteroidetes* and led to a reduction in secondary bile acid in cecal digesta and blood in these studied animals (Zarrinpar et al., 2018). Behr et al. (2019) compared antibiotic treatment from five different classes (lincosamides, glycopeptides, macrolides, fluoroquinolones, and aminoglycosides) in adult rats, and analyzed 20 primary and secondary bile acids in plasma and fecal samples of control and treated animals. They found that antibiotic treatment induced significant changes in bile acid profile in both plasma and feces, and these changes were dependent on antibiotics used. Altogether, these results suggested that different antibiotic treatments could have differing effects on gut bacteria composition and metabolism.

It has reported that the antibiotic-induced changes in gut microbiota might contribute to the inflammation responses through disrupt metabolism homeostasis, promoting the development of certain diseases such as obesity, liver disease, and diabetes (Qin et al., 2012; Korpela et al., 2016; Zhang et al., 2021a). Antibiotic-induced microbiome-depleted (AIMD) mice had a significant reduction of fecal short-chain fatty acids (SCFAs), especially butyrate, resulting in decreased baseline serum glucose level and improved insulin sensitivity (Fu et al., 2018). It has been shown that anti-inflammation metabolite SCFAs can exert beneficial effects against intestinal inflammation and protection for intestinal epithelial integrity (Murugesan et al., 2018). Vrieze et al. (2014) have shown that in male obese subjects, oral administration of vancomycin for 7 days reduced gut microbial diversity with a clear decrease in *Firmicutes* and a compensatory increase in *Proteobacteria*. Concomitantly, alternations in gut microbiota were predominantly associated with decreased fecal secondary bile acids, but with a simultaneous postprandial increase in primary bile acids in plasma, resulting in decreased peripheral insulin sensitivity. In mouse model, administration of antibiotics in the first 6 weeks of mice has caused increasing *Akkermansia* and decreasing *Bacteroidales* in gut microbiota, and concomitantly a decreased proportion of Treg and Th17 cells in the small intestinal lamina propria. These changes appeared to be responsible for the development of diabetes in adult mice (Livanos et al., 2016). These results suggested that different antibiotic treatments may have differing effects on metabolism and inflammation response, resulting in different disease outcomes. Recently, alteration in gut microbiome has been found to be associated with antibiotic tolerance in high-fat-diet-fed (HFD) mice (Liu et al., 2021). Compared with mice who were fed by a standard diet, mice with HFD had higher bacterial load. However, these bacteria with predominant *Lachnospiraceae* and *Muribaculaceae* families and fewer *Bacteroidaceae* and *Akkermansiaceae* families, concomitantly with substantially decreased level of indole-3-acetic acid (IAA) in fecal and serum samples, displayed lower susceptibility to antibiotic treatment. Moreover, the long-term antibiotic treatment for 62 days, rather than the short-term treatment for 3 days, could effectively reverse this HFD-induced antibiotic tolerance (Liu et al., 2021). These findings implied that the long-term antibiotic treatment in a HFD feeding of mice resulted in an alteration in the gut microbiota and subsequently changes in the levels of some specific metabolites, such as IAA, therefore reducing antibiotic efficacy against bacterial infections.

Collectively, it has established that in antibiotic-treated mice, the altered gut microbiome can affect host metabolic homeostasis and promote the development of multiple metabolic disorders. However, the short-term use of a single antibiotic does not seem to have such significant metabolic effects (Reijnders et al., 2016). Indeed, less is known about how long-term of antibiotic-induced disruption of gut microbiota influences host systemic metabolomes and immune responses and whether these changes are related to the type and timing of antibiotics used. The primary aim of this study was to investigate how long-term use of antibiotics induced gut dysbiosis together with dysmetabolism and their effects on host inflammatory response. The secondary aim was to compare the changes in gut microbiota composition, metabolic profiling, and levels of key cytokines caused by different antibiotics: vancomycin (VAN) and polymyxin B (PMB), which target gram-positive and gram-negative bacteria, respectively.

## Materials and Methods

### Ethics Statement

The animal experiments were approved by the Institutional Animal Care and the Animal Ethics Committee of Capital Medical University, Beijing, China and conducted in accordance with the guidelines set by the committees.

### Study Design

**Figure 1:**
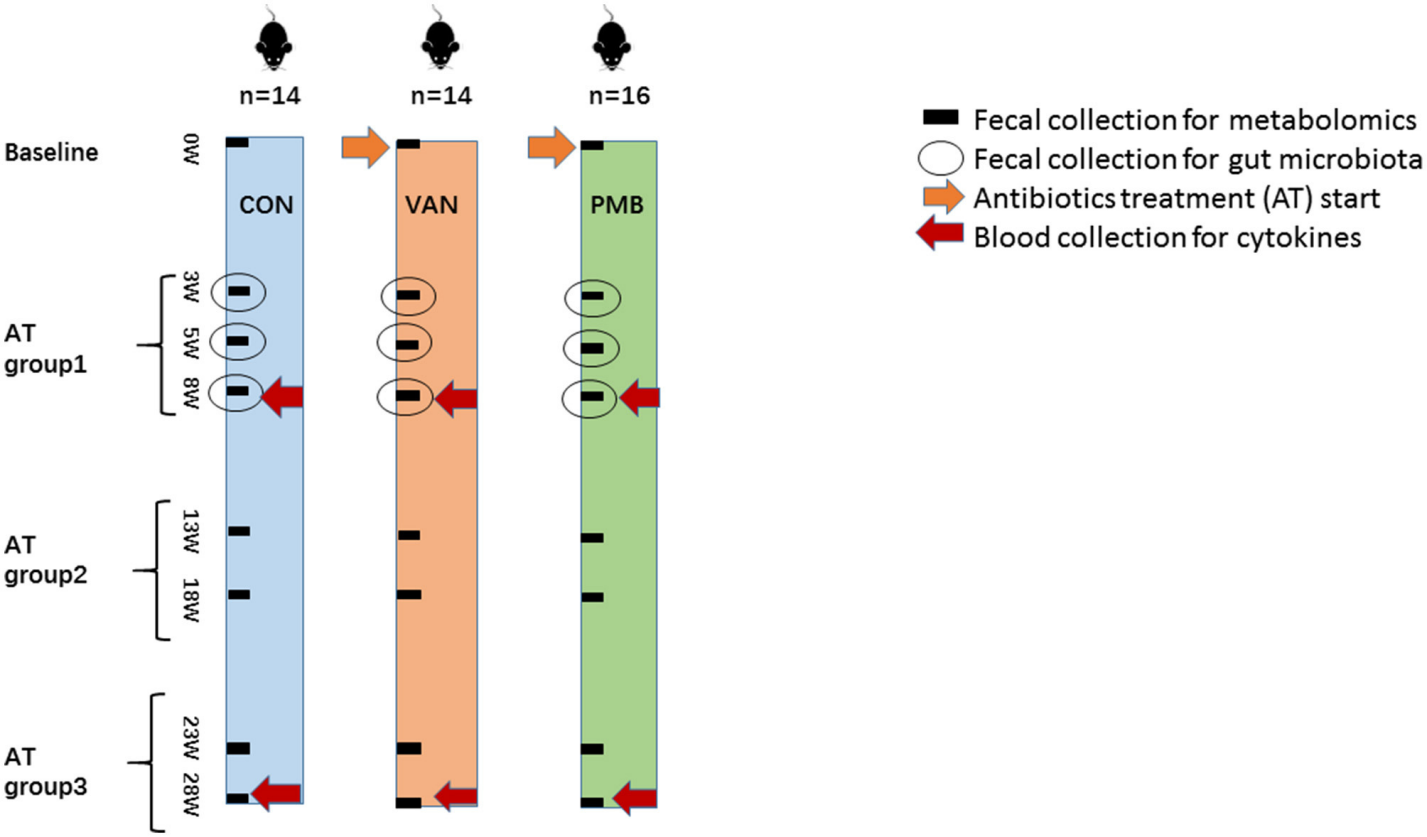
Scheme of the study design. A total of three groups of 3-week-old female C57BL/6 mice (*n* = 44) were continuously treated with vancomycin (VAN, *n* = 14), polymyxin B (PMB, *n* = 16), or water (CON, *n* = 14), respectively, for up to 28 weeks. The fecal samples before and after treatment for 3, 5, 8, 13, 18, 23, 28 weeks were used for metabolomic analysis. The fecal samples collected at 3-, 5-, and 8-week treatment were examined by 16S rRNA gene sequencing. Blood was collected after 8- and 28-week treatment for the determination of serum cytokines. Antibiotic treatment (AT) groups 1, 2, and 3 referred to the timing of treatment for 3, 5, and 8weeks, for 13 and 18 weeks, and for 23 and 28 weeks, respectively. AT, antibiotic treatment; VAN, vancomycin; PMB, polymyxin B; CON, control.

The 3-week-old female C57BL/6 mice, purchased from the Academy of Military Medical Sciences, Beijing, China, were first housed in the animal facility of the Capital Medical University for 1 week to acclimatize the environment. Mice were randomized into three groups, vancomycin-treatment group (VAN group, *n* = 14), polymyxin B-treatment group (PMB group, *n* = 16), and water treatment group as controls (CON group, *n* = 14). Mice were treated with VAN and PMB for up to 28 weeks. Antibiotics were administered in drinking water with concentrations of vancomycin (0.5 mg/ml) and polymyxin B sulfate (0.1 mg/ml) as previously described (Sun et al., 2019). CON group mice received sterilized water only. Water containers were changed one time a day to supply fresh antibiotics. During the treatment, water consumption was similar based on the daily observations. Body weight was monitored for each animal every week. In this study, antibiotic treatment (AT) groups 1, 2, and 3 referred to the timing of treatment for 3, 5, and 8 weeks, for 13 and 18 weeks, and for 23 and 28 weeks, respectively. The scheme of the study design is shown in Figure 1.

### Sample Collection

Before the treatment, baseline samples were collected. The fecal samples before and after treatment for 3, 5, 8, 13, 18, 23, and 28 weeks were used for metabolomic analysis. The fecal samples collected at 3-, 5- and 8-week treatment were also used for 16S rRNA gene sequencing. The blood samples were collected at 8- and 28-week treatment for the determination of cytokines. At 8- and 28-week treatment, mice were sacrificed and the colons were excised. The removed colon tissue was fixed in formalin. In addition, the collected fecal and plasma samples were frozen and stored at −80°C until processing.

### Hematoxylin–Eosin Staining

The intestinal histopathology was evaluated by hematoxylin–eosin (HE) staining. The distal colon was removed and washed using 0.9% saline. The colon tissue 1 cm proximal to the cecum was cut and then part of it was fixed in 4% formalin and embedded in paraffin for HE staining. The stained sections were observed and photographed under a light microscope (with 200× magnification).

### Genomic DNA Extraction and 16S rRNA Gene Sequencing

Bacterial genomic DNAs were extracted using the QIAamp Fast DNA Stool Mini Kit (Qiagen, Hilden, Germany) following the manufacturer's instructions. The V4–V5 regions of the prokaryotic 16S rRNA gene were amplified using the universal primer pair 515F (5′-GTGYCAGCMGCCGCGGTA-3′) and 909R (5′-CCCCGYCAATTCMTTTRAGT-3′) with barcode and then sequenced and analyzed (Ni et al., 2017). The reason to choose the V4–V5 regions was that we also wanted to detect low abundant bacteria in the fecal samples (Huang et al., 2018). The barcoded amplicons from all samples were normalized, pooled to construct the sequencing library, and then sequenced by an Illumina MiSeq to generate pair-ended 250 nt reads. Raw read data of all samples were deposited in the NCBI BioProject database under the accession number PRJNA788900.

The paired-end reads from the DNA fragments were merged using FLASH (v1.2.7) (Magoc and Salzberg, 2011). Sequencing data were analyzed using Quantitative Insights into Microbial Ecology (QIIME) 1.9.1 and R v3.3.1 (von Bergen et al., 2013). Operational taxonomic units (OTUs) were clustered with 97% sequence similarity using UPARSE (version 7.0.1001). The normalized OTU tables were used for diversity and statistical analyses. Bacterial diversity of the samples (alpha diversity) was calculated with observed species, Chao 1, ACE, Shannon, Simpson, and PD whole tree indexes (Christian et al., 2015). Structure of microbial communities (beta diversity) was calculated by weighted UniFrac distances (McMurdie and Holmes, 2013). Curtis similarity clustering analysis was used to perform a principal coordinate analysis (PCoA). The linear discriminant analysis (LDA) effect size (LEfSe) model was used to identify the differences in microbiota composition for phylotypes (Segata et al., 2011). Based on the normalized relative abundance matrix, taxa with significantly different abundances were determined by LEfSe using Kruskal–Wallis rank sum test.

### Metabolomic Detection and Data Analysis

The untargeted metabolomics by ultraperformance liquid chromatography coupled with quadrupole time-of-flight tandem mass spectrometry (UPLC Q-TOF MS) analysis was performed on Nexera X2 system (Shimadzu, Japan) coupled with a TripleTOF 5600 quadrupole-time-of-flight mass spectrometer (SCIEX, USA). Samples were separated on an Agilent ZORBAX Eclipse Plus C18 column (2.1 mm × 100 mm, 3.5 μm), maintained at 35°C, and the flow rate used was 0.5 ml/min. The mobile phase consisted of water (A) and acetonitrile (B) both containing 0.1% (v/v) formic acid. The gradient was initiated with 2% B for 1 min and then linearly increased to 90% B within 13 min. The gradient was kept at 90% B for 16 min and then back to 2% B within 0.1 min and held at 2% B for another 16 min. The total run was 20 min. Parameters of the UPLC Q-TOF MS were set as described previously (Zhu et al., 2019).

The raw data acquired by UPLC Q-TOF MS were imported to MarkerView software (version 1.2.1, SCIEX, USA) to conduct data pretreatment procedures. After the algorithm operation of the software, a peak table with retention time (t_R_), m/z value, and corresponding peak intensity was generated. MetaboAnalyst 4.0 (http://www.metaboanalyst.ca/MetaboAnalyst) was used for chemometric analysis, such as partial least squares discriminate analysis (PLS-DA). To identify the potential biomarkers, the accurate mass, retention time, and the fragmentation pattern from MS/MS were used. Human Metabolome Database (http://www.hmdb.ca/) was used to screen the potential biomarkers through m/z acquired by UPLC Q-TOF MS with molecular weight tolerance ± 10 ppm. In addition, Kyoto Encyclopedia of Genes and Genomes (KEGG) (http://www.kegg.jp/) were used to identify the metabolic pathways as described previously (Kanehisa and Goto, 2000).

### Determination of Plasma Cytokines

For the determination of inflammatory cytokines in plasma, a ProcartaPlex Mouse High-Sensitivity 6-plex Panel (cat. no. PPX-06) (Invitrogen, Vienna, Austria) for 6 cytokines, interferon-γ (IFN-γ), interleukin-2 (IL-2), IL-10, IL-13, IL-17A, and tumor necrosis factor-α (TNF-α), was designed for this study. The assays were performed on the MAGPIXTM platforms (Luminex, Austin, TX, United States) following the manufacturer's instruction. The range of detection for these cytokines was as follows: IFN-γ, 0.9–3,800 pg/ml; IL-2, 1.8–7,400 pg/ml; IL-10, 1.9–7,850 pg/ml; IL-13, 2.4–10,100 pg/ml; IL-17A, 1.4–5,950 pg/ml, and TNF-α, 3.1–13,000 pg/ml. IFN-γ was considered the signature cytokine of the Th1 cell; IL-13, the signature cytokines of the Th2 cell; IL-17A, the signature cytokine of the Th17 cell; TNF-α, the signature cytokine of proinflammatory; IL-10, the signature cytokine of anti-inflammation; and IL-2, the cytokine of T cell activation.

### Statistical Analysis

Data were analyzed using IBM SPSS statistics 24.0 software for Windows (IBM Corp., Armonk, NY, USA), GraphPad Prism 6.0 (La Jolla, CA, USA) software, and Image R software. Data were presented as mean values ± SEM for independent experiments. For comparison between groups, a paired *t*-test was performed. Multiple comparisons among groups were calculated by one-way analysis of variance (ANOVA). Correlations were performed using a non-parametric Spearman's test. Continuous variables were compared using the Wilcoxon rank sum test between the two groups. A two-sided *p* < 0.05 indicated a significant difference. The threshold logarithmic LDA score for discriminative features was set as 4. PLS-DA was used to determine the distributions and find the metabolic differences between two groups using MetaboAnalyst 4.0. The PLS-DA models were cross-validated using a 10-fold method with unit variance scaling. The parameter R^2^ was used to evaluate the fitting condition for the PLS-DA models, and Q^2^ was used to assess predictive ability. Metabolite data were log2-transformed for statistical analysis. The ions with variable importance in projection (VIP) values>1.2, fold change (FC)>1.5 or FC<0.5, and adjusted *p* < 0.05 were considered as potential differentially expressed metabolites.

## Results

### Effect of Antibiotic Treatment on Mouse Growth Performance and Gut Phenotype

**Figure 2:**
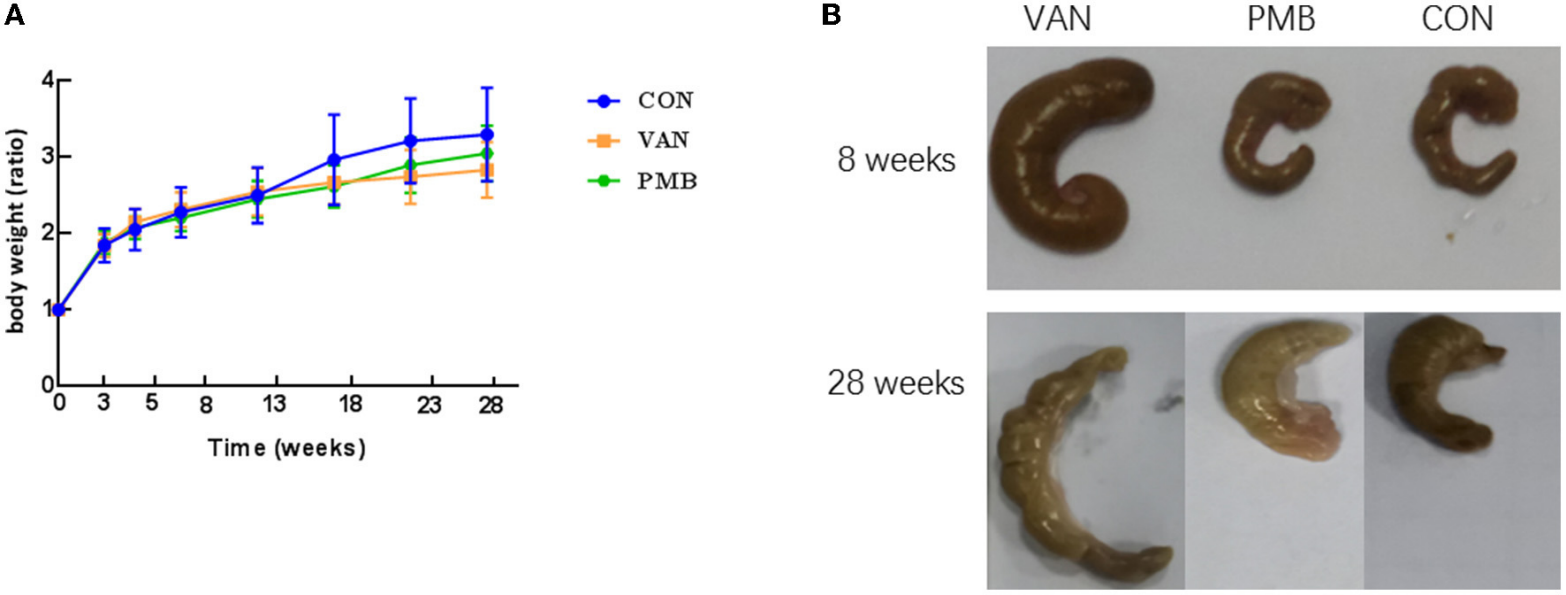
Effect of antibiotic treatment on mouse growth performance and gut phenotype. **(A)** Time curve of body weight ratio changes at different time points. **(B)** The caecum of mice with antibiotics-treated for 8 and 28 weeks. VAN, vancomycin; PMB, polymyxin B; CON, control.

The treatment by VAN or PMB did not affect the average body weight of the mice, when compared to control mice (all *p* > 0.05 at each time point, Figure 2A). However, a heavier caecum was observed in VAN-treated mice (Figure 2B). The histopathological results showed that the changes in microvilli, epithelium, and submucosal tissues in colon with VAN 8-week treatment. The inflammatory cell infiltration was observed in mice with VAN 28-week treatment (Supplementary Figure 1). Although there was no obvious structure change in mice with PMB 8-week treatment, the inflammatory cell infiltration was clearly observed. Changes in colon structures and tissues were also observed in mice with PMB 28-week treatment.

### Long-Term Use of Antibiotic-Induced Changes in Gut Microbiota

The average length of reads was 372 bp per sample (ranging from 213 to 410 bp). The average tags were 76, 194 per sample. Of them, 75, 455 were effective tags. Sequences with more than 97% similarity were assigned to the same OTUs. In total, 1,474 OTUs were detected.

**Figure 3:**
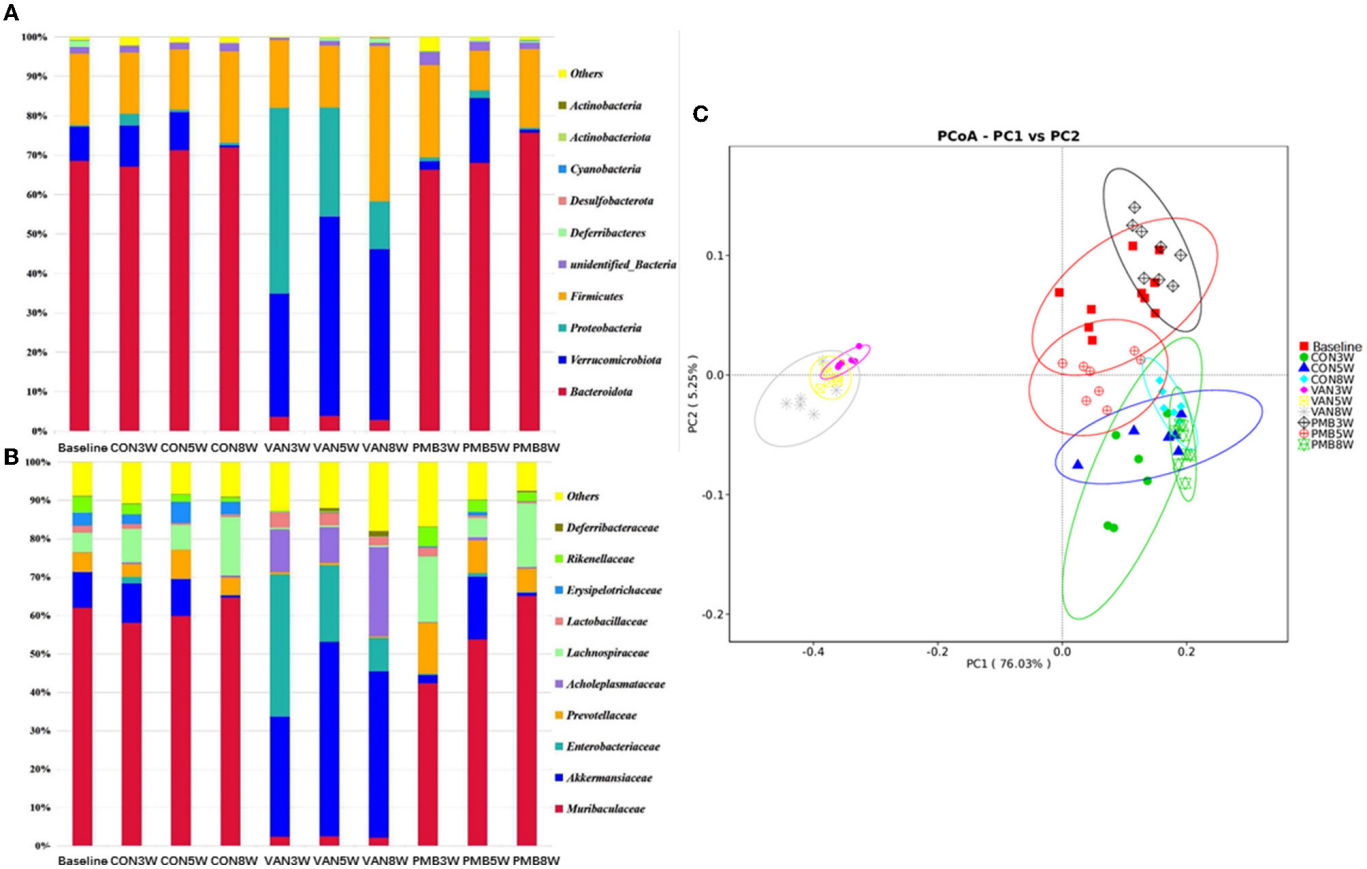
Antibiotic-induced composition changes of gut microbiota. Distribution of the predominant bacteria with antibiotics-treated at phylum level **(A)** and family level **(B)**. **(C)** Principal coordinate analysis (PCoA) of fecal samples in control and antibiotic-treated groups by Curtis similarity clustering analysis. VAN, vancomycin; PMB, polymyxin B; CON, control.

The control and PMB-treated mice had similar composition, with the most predominant phylum *Bacteroidetes* [relative abundance (RA): 68.5 and 70.0%] and *Firmicutes* (RA: 18.1 and 17.7%). For VAN-treated mice, phylum *Verrucomicrobia* and its family *Akkermansiaceae* and phylum *Proteobacteria* and its family *Enterobacteriaceae* were significantly increased in comparison to controls (Figures 3A,B). It is known that the overgrowth of *Enterobacteriaceae* might lead to release of large amounts of lipopolysaccharide (LPS). The function prediction analysis indicated that LPS biosynthesis was markedly enriched in VAN-treated mice. However, phylum *Bacteroidetes* and its family members *Muribaculaceae, Prevotellaceae, Erysipelotrichaceae*, and *Rikenellaceae* were markedly decreased in VAN-treat mice. *Acholeplasmataceae* family (of *Firmicutes* phylum) with low abundancies in control mice was enriched significantly after VAN treatment (RA: 0.1 vs. 14.5%, *p* = 0.002). PCoA clearly showed that VAN-treated mice had a district separation in gut microbiome composition from both PMB-treated and control mice (Figure 3C). These results suggested that VAN treatment, rather than PMB treatment, reshaped the whole intestinal ecology, by suppressing common commensals such as phylum *Bacteroidetes*, and facilitating the growth of the facultative anaerobic species of the phyla *Proteobacteria* and *Verrucomicrobia* that rarely occur in the normal condition.

**Figure 4:**
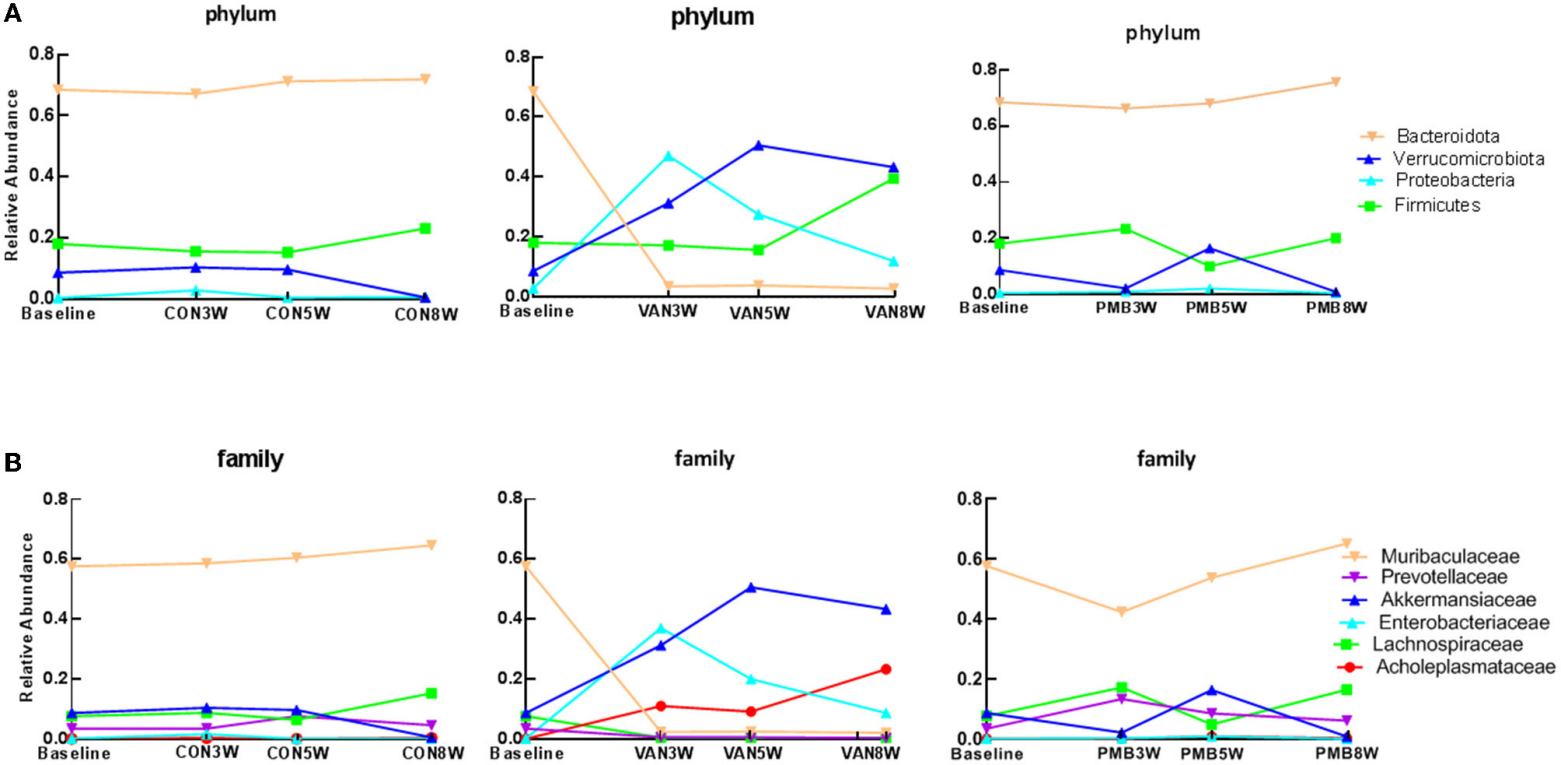
Antibiotic-induced dynamic changes in gut microbiota at phylum level **(A)** and family level **(B)**. The predominant taxa were indicated with different colors. VAN, vancomycin; PMB, polymyxin B; CON, control.

To explore dynamic change in gut bacteria during the treatment, the microbiota compositions after the treatment of 3, 5, and 8 weeks were analyzed. For controls, there was no observed compositional change between the baseline and three time points. Compared to the baseline, VAN-treated mice had a significant decrease in abundance of the phylum *Bacteroidetes* phylum after 3-week treatment and remained low at 5 and 8 weeks (baseline vs. 3-, 5-, and 8-week treatment: 68.5 vs. 3.6%, 3.8 and 2.8%, *p* = 0.002) as well as its family *Muribaculaceae* and *Prevotellaceae* (Figures 4A,B). In contrast, the abundances of the phyla *Verrucomicrobia* (7.4 vs. 31.3%, 50.6 and 43.4%, *p* = 0.003) and *Proteobacteria* (0.4 vs. 47.1%, 27.6 and 12.0%, *p* = 0.001) as well as their families *Akkermansiaceae* (7.4 vs. 31.3%, 50.6 and 43.4%, *p* = 0.003) and *Enterobacteriaceae* (0.2 vs. 35.5%, 20.1 and 8.7%, *p* = 0.002) were significantly increased after 3-week treatment and remained high at 5 and 8 weeks. The different taxa between baseline and VAN- or PMB-treated identified by LEfSe analysis are shown in Supplementary Figure 2. At genus level, correspondingly, *Akkermansia muciniphila* (7.4 vs. 31.3%, 50.6 and 43.4%, *p* = 0.003) and *Escherichia coli* (0.1 vs. 28.6%, 16.6 and 7.4%, *p* = 0.001) were markedly increased. Meanwhile, within PMB-treated 3-, 5-, and 8-week-old mice, a fluctuation around baseline of *Firmicutes* and *Verrucomicrobia* phylum and its family *Lachnospiraceae* and *Akkermansiaceae* were observed.

**Figure 5:**
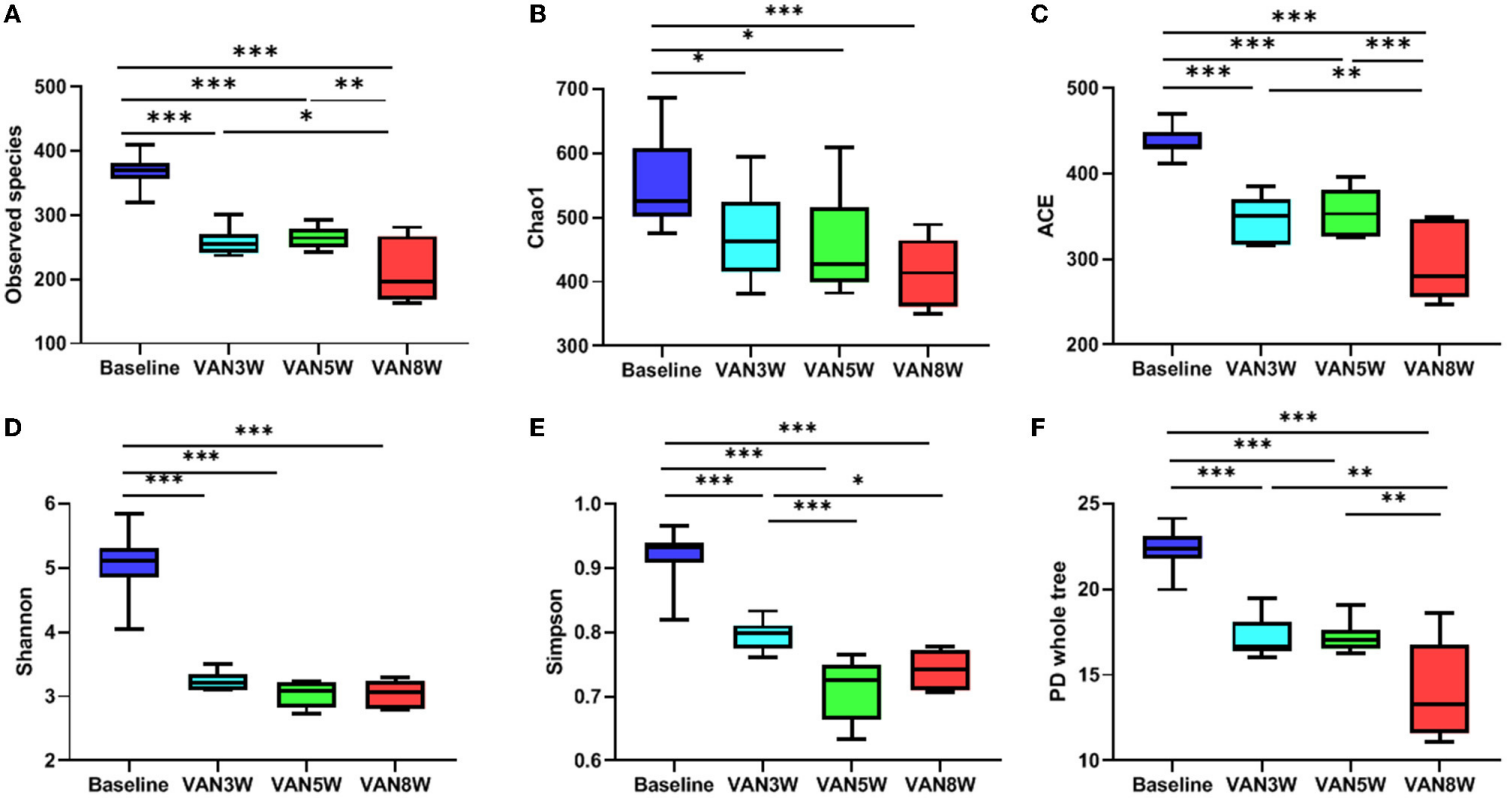
Vancomycin induced change in alpha diversity. Observed species, Chao 1, ACE, Shannon, Simpson, and PD whole tree indexes were shown in **(A–F)**, respectively. Significant differences between the two groups were marked, and *p < 0.05; **p < 0.01, and ***p < 0.001. VAN, vancomycin.

**Figure 6:**
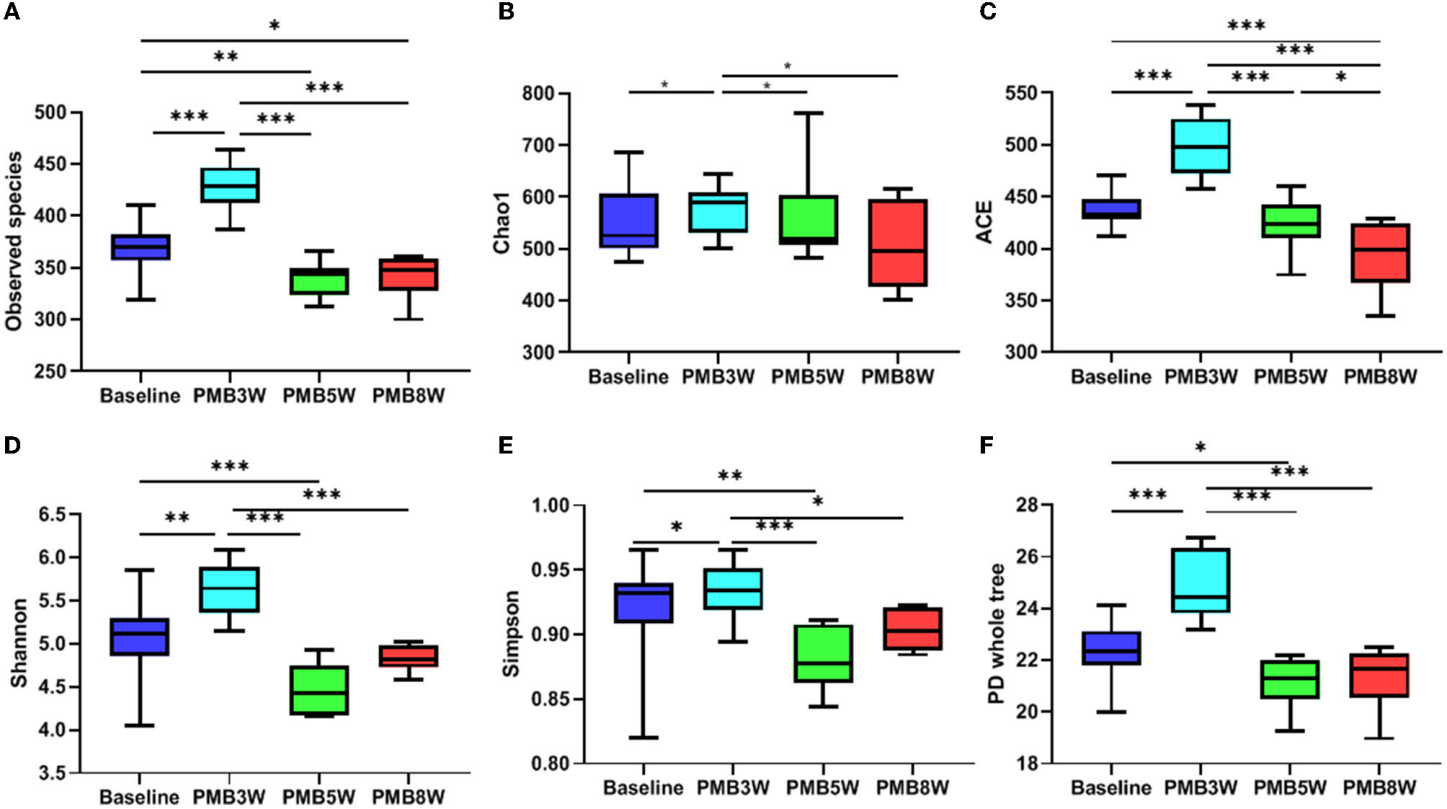
Polymyxin B induced change in alpha diversity. Observed species, Chao 1, ACE, Shannon, Simpson, and PD whole tree indexes were shown in **(A–F)**, respectively. Significant differences between the two groups were marked, and * *p* < 0.05; ** *p* < 0.01, and *** *p* < 0.001. PMB, polymyxin B.

Vancomycin -treated mice had significantly decreased diversity and richness of gut microbiota. Compared to baseline, all alpha diversity indexes in VAN-treated mice were significantly decreased (all *p* < 0.05, Figure 5). Moreover, indexes of observed species, ACE, Simpson, and PD whole tree were further decreased in mice after 8-week treatment by VAN in comparison with those after 3-week treatment (all *p* < 0.05). This was mainly due to a complete disappearance of the phylum *Bacteroidetes* and its families. For PMB-treated mice, all alpha -diversity indexes were increased after 3-week treatment, mainly ascribed to the increased richness of *Firmicutes* (all *p* < 0.05, Figure 6). After 5-week treatment by PMB, however, all alpha diversity indexes were significantly decreased when compared to those at 3 weeks (all *p* < 0.001), and some of these indexes were even lower than those detected at baseline. After 8-week treatment, these indexes remained more or less at the same levels as those at 5-week treatment.

### Long-Term Use of Antibiotics and Change in Metabolism

**Figure 7:**
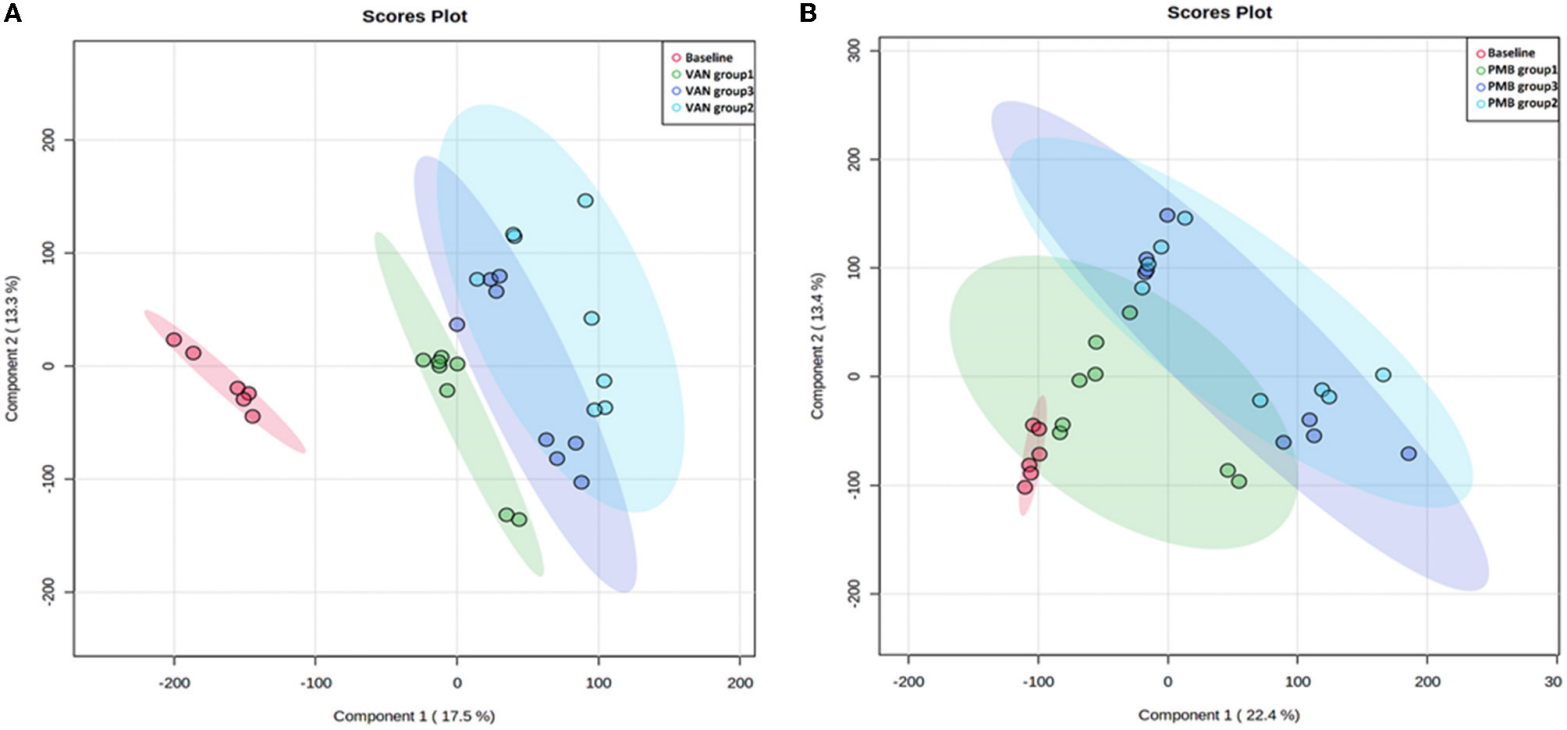
Vancomycin **(A)** and polymyxin B **(B)** induced metabolic profiling at different time points determined by partial least squares discriminate analysis (PLS-DA). VAN, vancomycin; PMB, polymyxin B; CON, control.

To improve the separation of different time points within the group and between the groups, we used PLS-DA to visualize the metabolic profiling differences. For VAN-treated mice, differences in metabolic profiles were well separated between baseline and VAN treatment groups 1–3 as well as among the VAN treatment groups 1–3 (Figure 7A). However, the differences were less obvious for PMB-treated mice (Figure 7B). Moreover, the metabolic profile of VAN AT group 1 was separated from that of VAN AT groups 2 and 3. There were some overlapping between VAN AT groups 2 and 3. However, the metabolic profiles between PMB AT groups 1, 2, and 3 were overlapped, especially between the groups 2 and 3. These results suggested that the major changes in antibiotic-induced metabolic profiling occurred rather fast, at least after 3 weeks in our study. However, the magnitude of the changes became less obvious with the time of antibiotic treatment.

**Figure 8:**
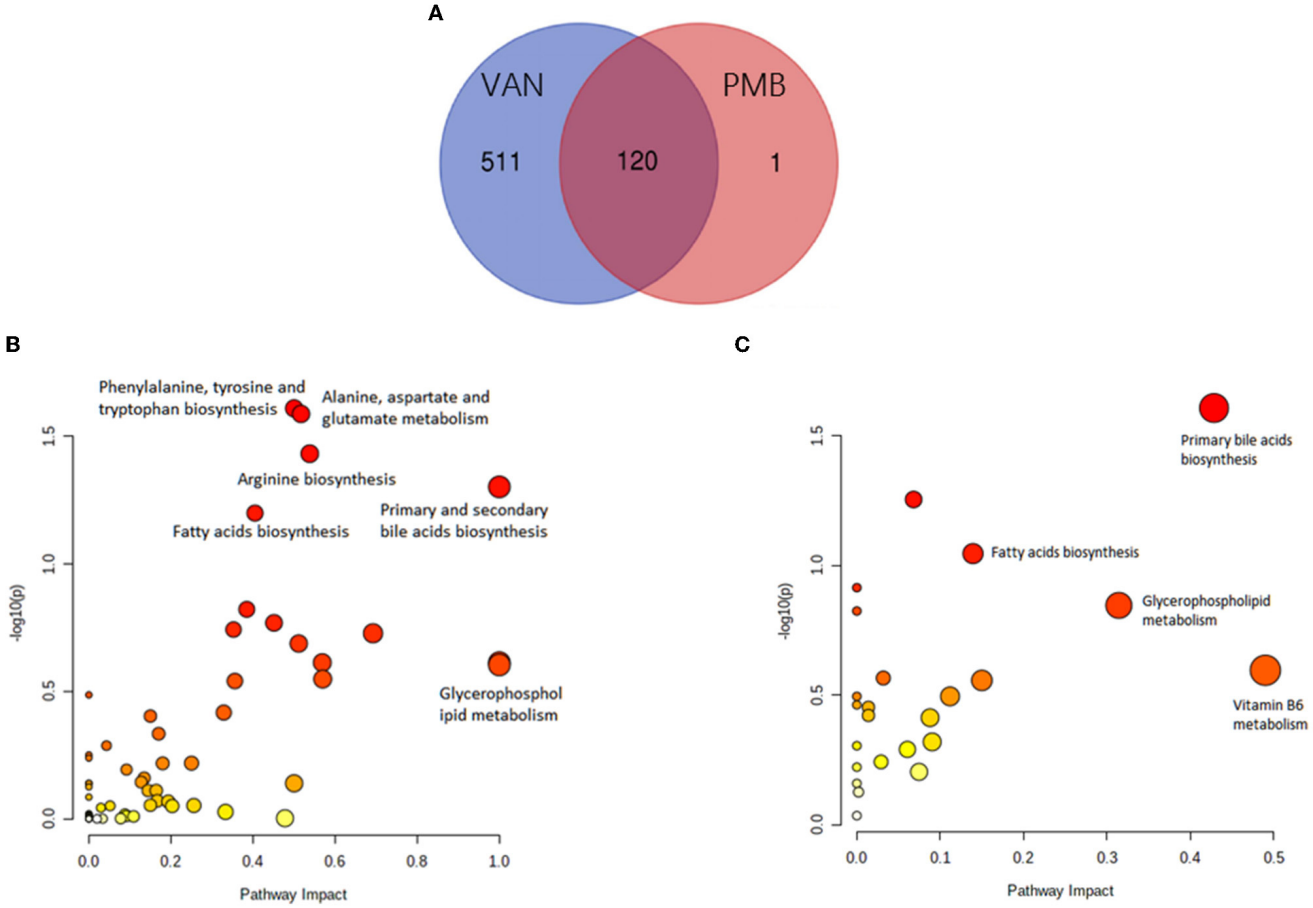
Antibiotic-induced changes in metabolites. **(A)** The number of metabolites induced by VAN and PMB treatment in comparison with the baseline. The VAN-**(B)** and PMB-**(C)** induced metabolites change enriched in significant KEGG pathway. VAN, vancomycin; PMB, polymyxin B.

Collectively, 54,161 positive- and negative-mode features were detected. Moreover, compared to baseline, a total of 631 and 121 different metabolites were identified in the VAN- and PMB-treated groups. Of these metabolites, 120 were in common (Figure 8A). Further analysis indicated that the 631 metabolites of VAN group were enriched in 59 KEGG pathway database, and of these pathways 6 became significant (*p* < 0.05, impact > 0.01). These pathways included primary and secondary bile acid biosynthesis; fatty acid biosynthesis; arginine biosynthesis; alanine, aspartate and glutamate metabolism; phenylalanine, tyrosine, and tryptophan biosynthesis; and glycerophospholipid metabolism (Figure 8B). The 121 metabolites of PMB group were enriched in 24 KEGG pathway database, of these pathways 4 became significant. They included primary bile acid biosynthesis; fatty acid biosynthesis; vitamin B6 metabolism, and glycerophospholipid metabolism (Figure 8C).

Compared to baseline, mice with VAN 3-week treatment had significant decrease in primary and secondary bile acids (BAs), except lithocholic acid (LCA). These changes remained unchanged up to 28 weeks. The decreased primary BAs included taurine, taurocholic acid (TCA), cholic acid (CA), glycocholic acid (GCA), and chenodeoxycholic acid (CDCA) (all *p* < 0.05). The decreased secondary BAs included deoxycholic acid (DCA), tauroursodeoxycholic acid (TUDCA), taurolithocholic acid (TLCA), isolithocholic acid, and hyodeoxycholic acid (HCA) (all *p* < 0.05). The VAN-induced changes in key metabolites involved in metabolic pathways are summarized in Supplementary Table 1 and Supplementary Figure 3. PMB 3-week treatment induced only primary BAs changed, a decrease of taurine (FC = 0.48, *p* = 0.04) and TCA (FC = 0.40, *p* = 0.01) and an increase of GCA (FC = 2.02, *p* = 0.023) and CDCA (FC = 1.98, *p* =0.026). PMB-induced changes in key metabolites involved in metabolic pathways are summarized in Supplementary Table 2 and Supplementary Figure 4.

Compared to baseline, VAN-treated mice also had a significant decrease in SCFAs and LCFAs. These SCFAs included acetic acid (FC = 0.06, *p* < 0.001), isobutyric acid (FC = 0.25, *p* = 0.002), and isovaleric acid (FC = 0.31, *p* = 0.004). Hence, VAN treatment obviously changed the SCFAs pool, most notably by decreasing acetic acid to undetectable levels. However, PMB-treated mice only had a lower concentration of acetic acid (FC = 0.48, *p* = 0.022). Moreover, VAN treatment induced significantly decreased levels of LCFAs including dodecanedioic acid, behenic acid, hexadecanedioic acid, arachidonic acid, 2-hydroxyhexadecanoic acid, heptadecanoic acid, prostaglandin E2, stearic acid, capric acid, palmitoleic acid, ricinoleic acid, and fumaric acid (all *p* < 0.05). However, there was no obvious change in LCFAs of PMB-treated mice. In addition, for PMB-treatment group and VAN-treatment group, all changes observed remained unchanged up to 28-week treatment.

Vancomycin treatment also induced significantly increased level of lysophosphatidylcholine (LPC). The increased LPCs included LPC (16:0), LPC [16:1(9Z)], LPC (18:0), LPC [18:1(9Z)], LPC [18:2 (9Z and 12Z)], LPC (O-18:0/0:0), and LPC [20:4 (5Z, 8Z, 11Z, and 14Z)] (all *p* < 0.05). However, PMB treatment only had an increase of LPC (14:0) and LPC [20:3 (5Z, 8Z, and 11Z)], and the difference was only significant in 28-week treatment (*p* = 0.003 and 0.004). VAN treatment also induced decreased level of phosphatidylcholine (PC) (all *p* < 0.05).

Vancomycin -induced metabolism pathway of amino acids was characterized with significant decrease of L-arginine, L-proline, L-tyrosine, dopamine, and L-dopamine as well as with increase of 5-hydroxy-L-tryptophan, beta-alanine, L-glutamic acid, and L-aspartic acid. Level of L-aspartic acid was significantly increased 188-folds compared to baseline. Level of L-glutamic acid was increased 5-folds, which correlated with most of the annotated top KEGG pathways, such as arginine biosynthesis; alanine, aspartate, and glutamate metabolism; arginine and proline metabolism; histidine metabolism; taurine and hypotaurine metabolism; D-glutamine and D-glutamate metabolism; and glutathione metabolism. PMB-induced vitamin B6 metabolism was characterized with a decreased pyridoxal.

### Relationship of Serum Metabolomic Profiling With Changed Gut Microbiota

**Figure 9:**
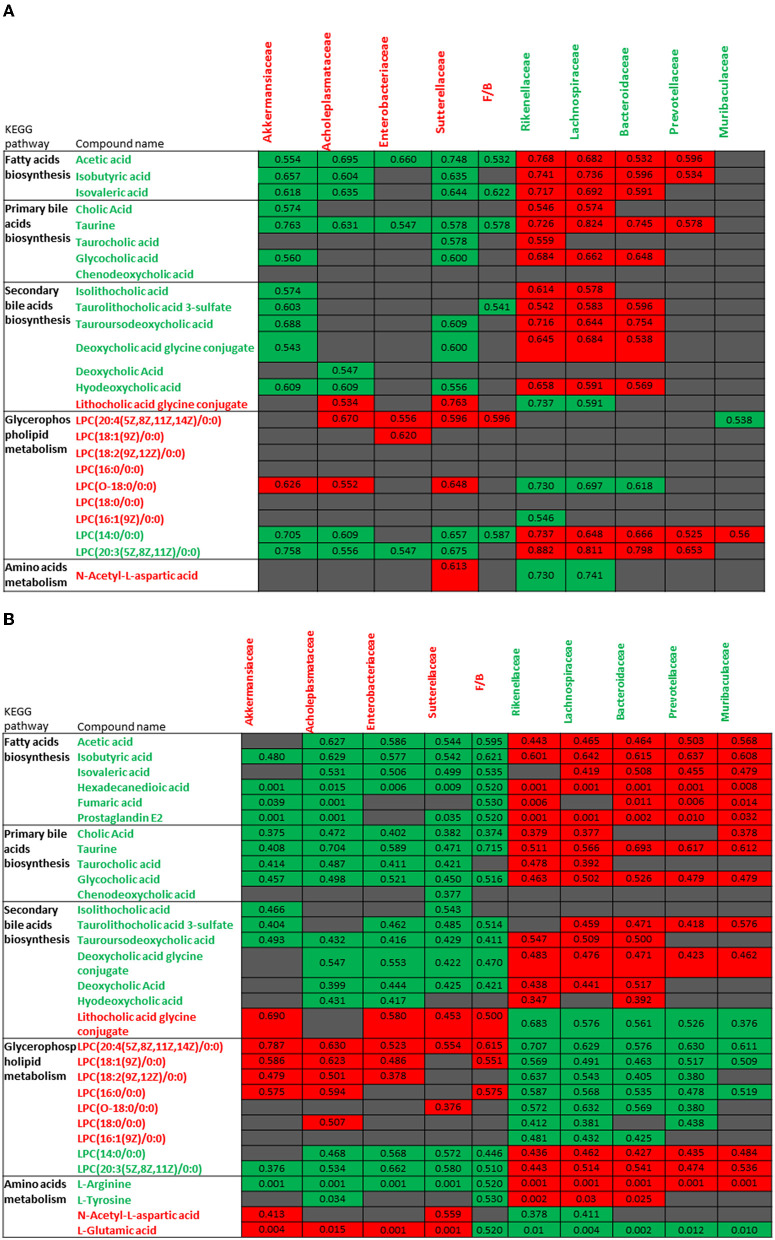
Correlations between the changes in metabolites of vancomycin treatment group 1 **(A)** and vancomycin treatment groups 2 and 3 **(B)** and abundances in different bacterial families. The number in color present Spearman's correlation coefficient: red color indicates positive correlation; green color indicates negative correlation.

Metabolomic profiles observed in VAN AT groups 1, 2, and 3 were correlated with changed gut microbiota, and similar pattern in these correlations was noticed (Figures 9A,B). The decreased levels of bile acids, fatty acids, L-arginine, and L-tyrosine were negatively associated with the increased abundances of *Akkermansiaceae, Enterobacteriaceae, Acholeplasmataceae*, and *Sutterellaceae* families as well as with increased ratio of *Firmicutes* to *Bacteroidetes*. However, these decreased levels of metabolites were positively correlated with decreased abundances of *Rikenellaceae, Lachnospiraceae, Bacteroidaceae, Prevotellaceae*, and *Muribaculaceae* families. Furthermore, the increased levels of LPCs, L-glutamic acid, and L-aspartic acid showed the positive correlations with above 4 increased families as well as with increased ratio of *Firmicutes* to *Bacteroidetes*, and negative correlation with above 5 decreased families after VAN treatment. In contrast, there was no obvious correlation between PMB-induced metabolic change and altered gut microbiota.

### Antibiotic-Induced Systemic Inflammatory Cytokines

**Figure 10:**
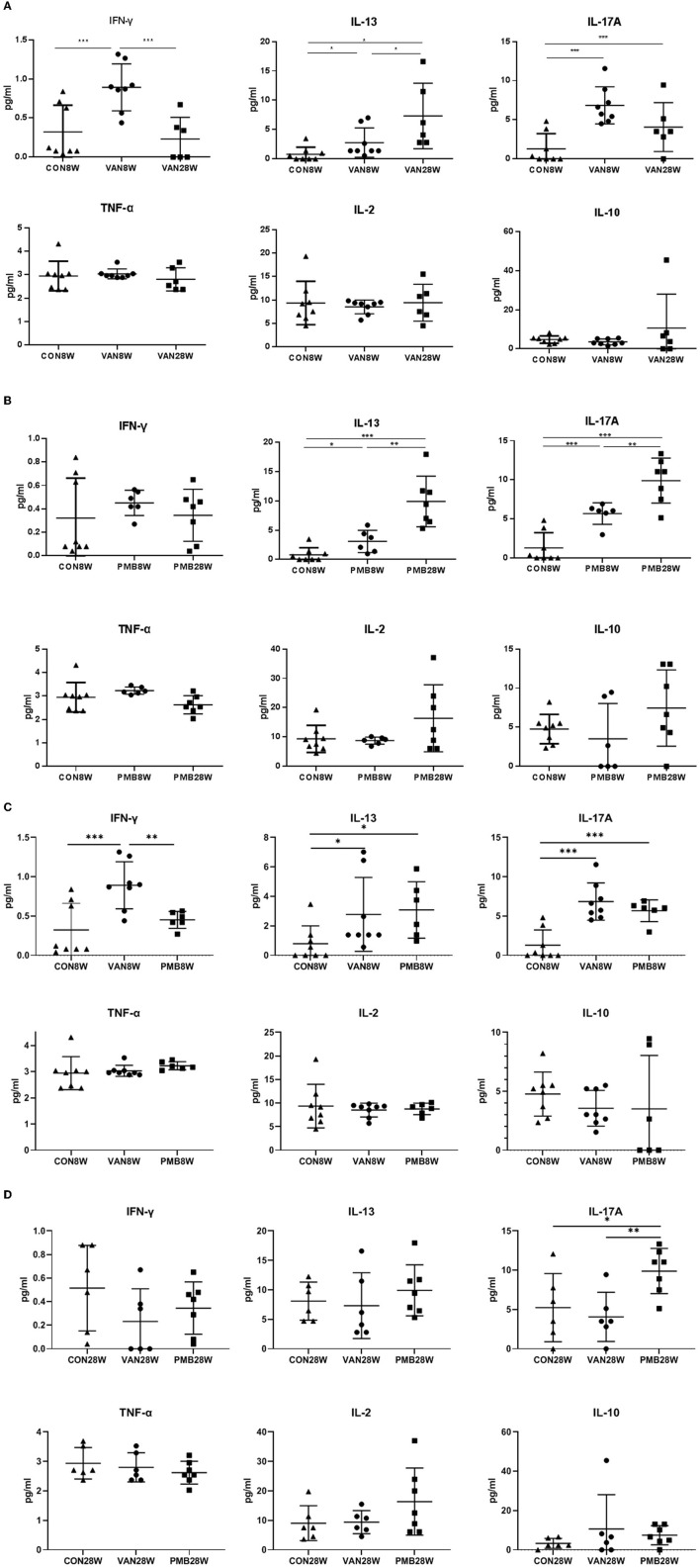
Changes in levels of serum cytokines after vancomycin treatment for 8 and 28 weeks **(A)**, polymyxin B treatment for 8 and 28 weeks **(B)**, vancomycin and polymyxin B treatment for 8 weeks **(C)** and 28 weeks **(D)**. Significant differences between the two groups were marked, and *indicates *p* < 0.05; **p < 0.01 and ***p < 0.001.

First, we compared the levels of all cytokines between 8 and 28 weeks in the control mice. Among the 6 cytokines analyzed, no significant differences were found in 4 cytokines (IL-2, IL-10, IFN-γ, and TNF-α). For IL-13 and IL-17A, significant increase was observed at 28 weeks compared to 8 weeks (IL-13: 8.09 vs. 0.80 pg/ml, *p* = 0.011) and (IL-17A: 5.25 vs. 1.29 pg/ml, *p* = 0.040). We next compared the levels of cytokines between control mice and mice with VAN or PMB treatment at 8 and 28 weeks. Mice with 8-week VAN treatment had significantly higher level of serum IFN-γ (*p* = 0.001), IL-13 (*p* = 0.048), and IL-17A (*p* = 0.001), when compared to 8-week-old control mice. However, the differences in the three cytokines as well as other three cytokines were not observed between 28-week-old control mice and 28-week VAN treatment mice. When compared to VAN 8-week-old mice, VAN 28-week-old mice had higher levels of IL-13, but lower level of IFN-γ and IL-17A (Figure 10A). Mice with 8-week PMB treatment had significantly higher level of serum IL-13 (*p* = 0.043) and IL-17A (*p* =0.001), when compared to those observed in control mice at 8 weeks. However, 28-week PMB treatment mice only had higher level of IL-17A (*p* = 0.021) compared to 28-week-old control mice. When compared to PMB 8-week-old mice, PMB 28-week-old mice had significantly higher levels of IL-13 and IL-17A (Figure 10B). Compared to PMB 8-week treatment, VAN 8-week treatment had significantly higher level of serum IFN-γ (*p* = 0.006) (Figure 10C). Compared to VAN 28-week treatment, PMB 28-week treatment had higher level of IL-17A (*p* = 0.008) (Figure 10D). These results suggested that different antibiotic treatments may have differing effects on host immune responses, and the effects are likely related to the type, rather than timing of antibiotic used.

## Discussion

Exposures to antibiotics in both animal experiments and human studies have been shown to disrupt metabolism homeostasis and to promote metabolic disorders (Zhu et al., 2019). To the best of best knowledge, this study was the first to show that long-term antibiotic-induced gut dysbiosis contributes to host inflammatory responses through dysfunctional metabolism. With the long-term treatment, the gut bacteria could become tolerant to antibiotics to certain extent. Although there was no obvious change in gut microbiota and metabolites after 8-week treatment, our findings confirmed that antibiotic treatment up to 8 weeks can change both host gut microbiota and metabolism. Moreover, these changes observed in gut microbiota and subsequent host metabolism and inflammatory responses are likely related to antibiotic type rather than timing of antibiotics used. In addition, alternations in gut microbiota and subsequent change in metabolomic profiles take place rather fast after the use of antibiotics.

In this study, we found that the host responses to the treatment by distinct antibiotics were different. VAN treatment dramatically and rapidly altered the microbiota composition and diversity, whereas PMB-induced alternations seemed to be mild and even transient. In addition, the alternations observed in microbiota composition and diversity could recover to certain extent and even maintain for a long time in spite of continuous antibiotic treatment. This could be explained by the unchanged metabolomic profiles observed after the 8-week treatment. The immediate response of VAN-treated mice had far fewer bacterial sequences from the *Bacteroidetes* phylum, ascribed to decrease of diversity and richness. Instead, their microbiome had a compositional shift to phylum *Proteobacteria* and its family *Enterobacteriaceae*, phylum *Verrucomicrobia*, and its family *Akkermansiaceae*. In contrast, after the PMB treatment was started, mice had an increased *Firmicutes*. However, the increase stayed only for a short period, and its diversity was then decreased. This finding was in line with the result reported by the recent studies (Sun et al., 2019; Ran et al., 2020). It is known that the phylum *Bacteroidetes* is comprised of gram-negative and obligate anaerobic bacteria that constitute a major part of the normal flora in gastrointestinal tract. In addition, *Firmicutes* is mainly comprised of gram-positive clostridia (Thomas et al., 2011). Interestingly, our results showed that with relative long-term use of the antibiotic, VAN, rather than PMB, induced a “complete” disappearance of gram-negative *Bacteroidetes*. One potential explanation for the magnitude and duration of antibiotic effects *in vivo* could be the remarkable interdependence of different bacterial taxa in the gut. Additionally, previous study has revealed that gram-negative commensals could be depleted by vancomycin (Ubeda et al., 2010).

Several studies have confirmed that antibiotic treatment can induce profound changes in bile acid metabolism (Sayin et al., 2013; Vrieze et al., 2014). Primary bile acids, such as cholic acid and chenodeoxycholic acid, are produced in the liver from cholesterol by the enzyme cholesterol 7a-hydroxylase. Prior to their secretion into the small intestine, bile acids are conjugated with either taurine or glycine. Subsequently within the intestine, microbiota further convert primary bile acids into secondary ones (Begley et al., 2006). It has been shown that primary bile acids can be de-conjugated by bile salt hydrolases (*Bsh*) and bile acid-inducible (*Bai*) genes which are produced mainly by the taxa of *Bacteroidetes* and its families *Bacteroidaceae* and *Rikenellaceae* (Jiang et al., 2015; Gu et al., 2017). In our study, due to deficiency of the family *Bacteroidaceae* bacteria, antibiotic-treated mice had a decreased amount of secondary bile acids in particular. In addition, the reduction in primary and secondary bile acids may also partially ascribe to the enrichment of *Akkermansiaceae* with its metabolite of L-aspartate. *Akkermansiaceae* belongs to the phylum *Verrucomicrobia*, comprising only one member known as *Akkermansia muciniphila*. *A. muciniphila* is an abundant member in human intestinal microbiota and is an important bacterium in host adaptive immune response (Ansaldo et al., 2019). Several studies have shown the beneficial use of *A. muciniphila* to protect or prevent the worsening of metabolic disorders, such as obesity and metabolic dysfunction-associated fatty liver disease (Depommier et al., 2019; Zhang et al., 2021b). A recent study also reported that with 6-week gavage of *A. muciniphila* mice had increased expressions of bile acid reflux regulatory genes and decreased expressions of bile acid synthesis-related genes in gut (Rao et al., 2021). Meanwhile, these mice also had significantly increased L-aspartate levels in both fecal and plasma samples. Moreover, with the gavage of L-aspartate, mice also had markedly activated a network of genes involved in bile acid reflux and transportation in mouse colon. Thus, the richness of L-aspartate mediated by *Akkermansiaceae* and the deficiency of *Bacteroidetes* in the gut would provide a reasonable explanation for decreased levels of bile acids observed in our current study.

Jin et al. studied the effects of penicillin G, erythromycin, and their mixture on gut microbiota in mice and found that exposure to these oral antibiotics induced gut microbiota dysbiosis which was associated with chronic “low-grade” inflammation (Jin et al., 2016). Their finding was confirmed by our study results in which the levels of serum inflammatory cytokines after 8- or 28-week treatment by antibiotics were similar but all were higher than those observed in control mice. Further, increased or decreased levels of metabolites which were related to inflammatory or anti-inflammatory responses may together underlie the low-grade Inflammation induced by antibiotics. As *Bacteroidetes* exist as a group of gram-negative intestinal bacteria, their absence may imply a continuous death of these gram-negative bacteria in the gut. In our study, the absence of *Bacteroidetes* and predominance of *Enterobacteriaceae* for the antibiotic exposure period could be an important source of LPS. Previous study has shown that the LPS derived from gut bacteria can induce a chronic subclinical inflammatory process through LPS-LBP (lipopolysaccharide-binding protein) signaling pathway (Saad et al., 2016). Moreover, it was reported that vancomycin- and polymyxin B-induced dysmetabolism could increase intestinal permeability and facilitate LPS and microbial translocation (Feng et al., 2019; Ran et al., 2020). In addition, recent studies also illustrated that the concentration of LPC was positively correlated with *Enterobacteriaceae* family, together with increased gut permeability by damaging tight junction of colonic epithelial cells and promoting the release of proinflammatory cytokines (Riederer et al., 2011; Chang et al., 2017; Tang et al., 2021). LPC (16:0), LPC (18:0), LPC (18:1), LPC (18:2), and LPC (20:4) are the most abundant LPC in human. Indeed, we have observed the elevated levels of aforementioned LPCs in the fecal samples. We also found significantly increased level of L-glutamic acid in testing samples. It has been shown that L-glutamic acid can induce high expression of proinflammatory factors and low expression of tight junction protein [46]. Collectively, metabolite-related inflammation, together with the increased intestinal permeability, may contribute to the systemic inflammation.

In this study, we also observed a significant reduction in SCFAs of fecal samples after vancomycin and polymyxin B treatment. SCFAs are anti-inflammatory metabolites, which is known not only to inhibit the macrophage-dependent intestinal local inflammation that leads to the production of proinflammatory cytokines, but also can polarize systemic T cell responses (Scott et al., 2018). Moreover, SCFAs altered the metabolic behavior of macrophages to increase oxidative phosphorylation and promoted alternative macrophage activation. In addition, butyrate has been shown to decrease LPS translocation in intestines, thus reducing LPS-mediated inflammation (Hartstra et al., 2015). Another study also showed that antibiotic-induced long-term changes with SCFAs depletion were associated with intestinal barrier damage and increased epithelial permeability (Holota et al., 2019). Since the decrease in SCFAs could increase intestinal permeability, the change could also have a comprehensive effect on immune response. In addition, tryptophan and its metabolites such as kynurenic acid, serotonin, and quinolinic acid can also exert anti-inflammatory effects and decreased severity of dextran sodium sulfate (DSS)-induced colitis in mice (Etienne-Mesmin et al., 2017; Maerz et al., 2018). The other metabolite, L-arginine, has proven to inhibit inflammatory response and oxidative stress induced by LPS (Qiu et al., 2019). In our study, these metabolite-related anti-inflammatory response was also significantly decreased after the antibiotic treatment.

There were several limitations in this study. First, we did not reveal the direct mechanism of altered metabolic profiling in promoting inflammation in the antibiotic treatment mouse model. To achieve this, serum metabolomics should be performed in the future. Second, the gut microbiota of mice after 8-week treatment was not performed. However, metabolomic analysis was done in fecal samples collected at all study time points. The results obtained indicated that the change of metabolism occurred within 8 weeks after the treatment started. It is known that microbial changes occur faster than metabolism changes. Therefore, earlier time points within 8 weeks for 16S sequencing were done. Third, due to the limitation in sensitivity of 16S rRNA sequencing, the analysis can only reach to family and genera of microbiota, rather than species. Therefore, it should be kept in mind that the result of species identified in this study should be interpreted with caution. In addition, the intake of antibiotics used in drinking water by individual mouse was not quantified. However, the following findings obtained in our study may verify that the individual intake was similar during the intervention period: (1) the water consumption was similar based on the daily observations; (2) the treatment by VAN or PMB did not significantly affect the average body weight of the mice when compared to control mice; (3) similar bacterial composition was observed in individual mouse in the same group at the different time points in this study as well as in our previous study using the same design (Sun et al., 2019); (4) a heavier caecum and more inflammatory cell infiltration were observed in VAN-treated mice, compared to PMB and CON group, and (5) there was a significant difference of bacterial composition, metabolism, and inflammatory response between the three groups as well as at different time-point groups. Although the two antibiotics, vancomycin and polymyxin B used in this study, are not commonly used in clinical settings, polymyxin has been considered as a “last line of effective drug” for the treatment of carbapenem-resistant gram-negative bacterial infections (Zhang et al., 2021c).

## Conclusions

This study showed that the antibiotic-induced alterations in gut microbiota contribute to host inflammatory responses through the change in metabolic status, which are likely related to the type, rather than timing of antibiotic used. Alternations in gut bacteria and their subsequent metabolites can have a comprehensive effect on “low-grade” inflammation during/after the long-term antibiotic treatment.

## Data Availability Statement

The datasets presented in this study can be found in online repositories. The names of the repository/repositories and accession number(s) can be found in the article/Supplementary Material.

## Ethics Statement

The animal study was reviewed and approved by the Institutional Animal Care and the Animal Ethics Committees of Capital Medical University.

## Author Contributions

QH and NZ conceptualized and designed the study, prepared the original draft, and wrote and reviewed the manuscript. JL, NZ, NC, ZC, and FG performed animal experiments, sample collection, and data acquisition. NZ, JL, and QH analyzed and interpreted the data. All authors read and agreed to the published version of the manuscript.

## Funding

This study was supported by the National Key Technologies R&D Program for the 13th 5-year Plan (2017ZX10202101-004), the Beijing Natural Science Foundation and Haidian Innovation Joint Project (19L2043), and the fund of National Microbial Resource Center (No. NMRC-2021-2) and National Pathogen Resource Center (NPRC-32). The funding bodies had no role in the study design, data collection, data analysis or interpretation, or writing of the report.

## Conflict of Interest

The authors declare that the research was conducted in the absence of any commercial or financial relationships that could be construed as a potential conflict of interest.

## Publisher's Note

All claims expressed in this article are solely those of the authors and do not necessarily represent those of their affiliated organizations, or those of the publisher, the editors and the reviewers. Any product that may be evaluated in this article, or claim that may be made by its manufacturer, is not guaranteed or endorsed by the publisher.

## Supplementary Material

Supplementary Figure 1 The histopathology results of colon tissues treated by antibiotics for 8 and 28 weeks (HE stain, X 200). VAN, vancomycin; PMB, polymyxin B; CON, control.

Supplementary Figure 2 The histogram of the linear discriminant analysis (LDA) scores for differentially abundant taxa with baseline and VAN treated **(A)** and PMB treated **(B)**. Only taxa achieving an LDA significant threshold ≥ 4 are shown. VAN, vancomycin; PMB, polymyxin B.

Supplementary Figure 3 Vancomycin-induced changes in key metabolites, including primary bile acids **(A)**, secondary bile acids **(B)**, short-chain fatty acids **(C)**, long-chain fatty acids **(D)**, amino acids **(E)**, lysophosphatidylcholine **(F)**, and phosphatidylcholine **(G)**.

Supplementary Figure 4 Polymyxin B-induced changes in key metabolites, including primary bile acids **(A)**, short-chain fatty acids **(B)**, long-chain fatty acids **(C)**, lysophosphatidylcholine **(D)**, and phosphatidylcholine **(E)**.

Supplementary Table 1 Vancomycin-induced changes in key metabolites involved in metabolic pathways at different time points.

Supplementary Table 2 Polymyxin-induced changes in key metabolites involved in metabolic pathways at different time points.
