# Supplementary material for: Integrated Analysis of the Alterations in Gut Microbiota and Metabolites of Mice Induced After Long-Term Intervention With Different Antibiotics
Source: Front Microbiol. 2022 Jun 29;13:832915. doi: 10.3389/fmicb.2022.832915 (PMC9277126; doi:10.3389/fmicb.2022.832915)
Supplement: Supplementary Figure 1 — The histopathology results of colon tissues treated by antibiotics for 8 and 28 weeks (HE stain, X 200). VAN, vancomycin; PMB, polymyxin B; CON, control. [file Data_Sheet_1.zip › Supplementary Figures.PPTX]

## Slide 1
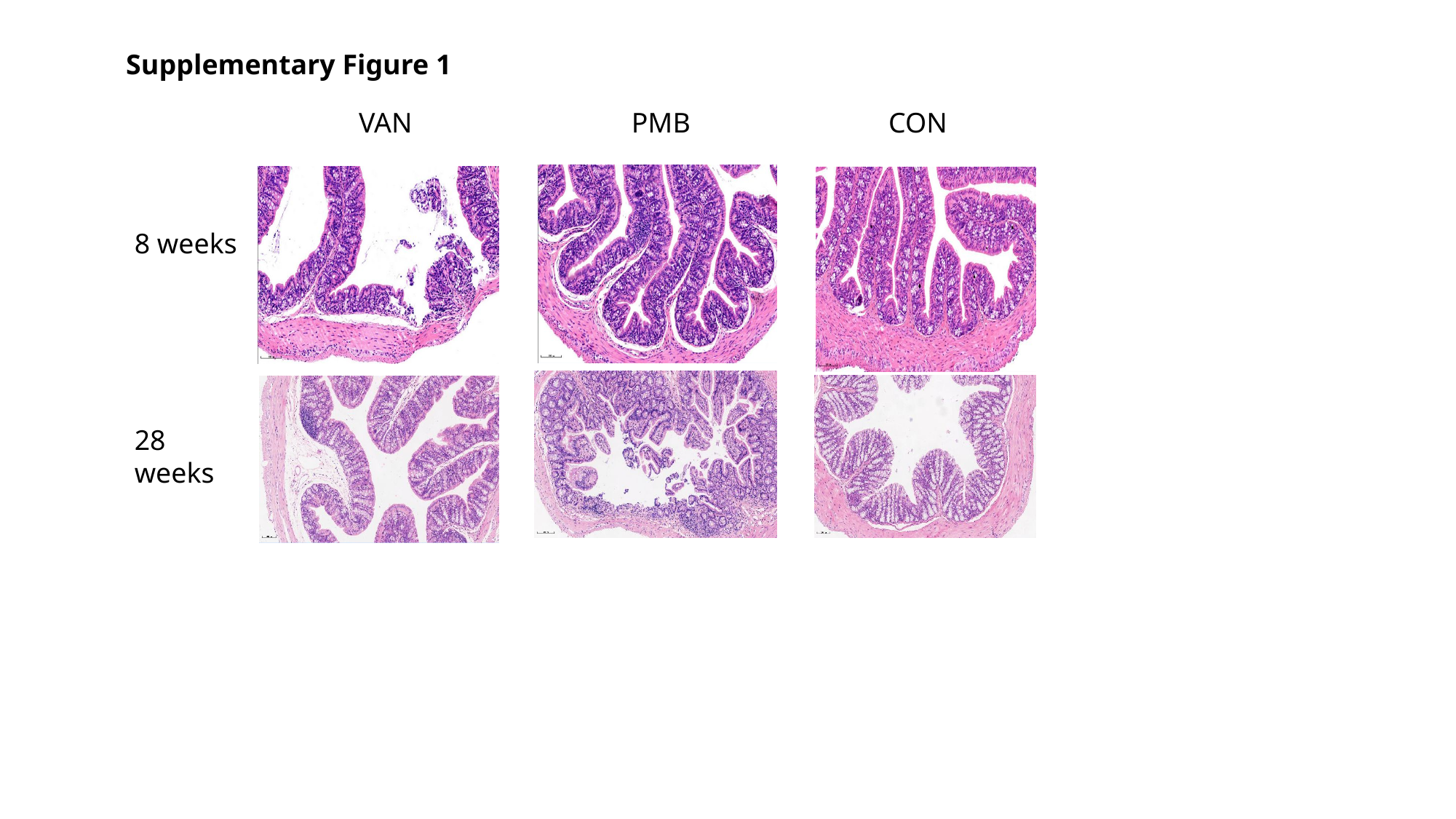

Supplementary Figure 1
 VAN PMB CON
8 weeks
28 weeks

## Slide 2
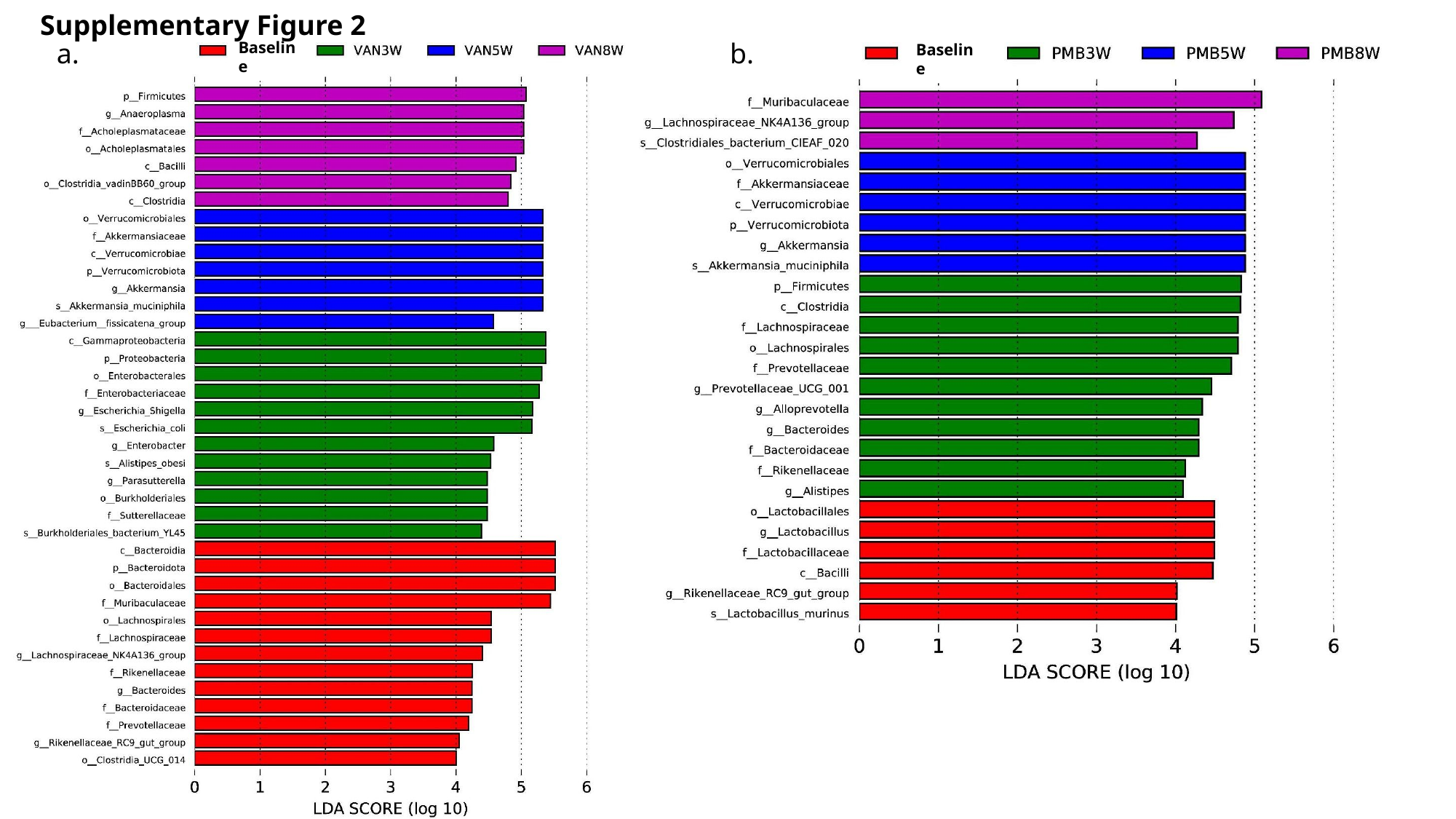

Supplementary Figure 2
a. b.
Baseline
Baseline

## Slide 3
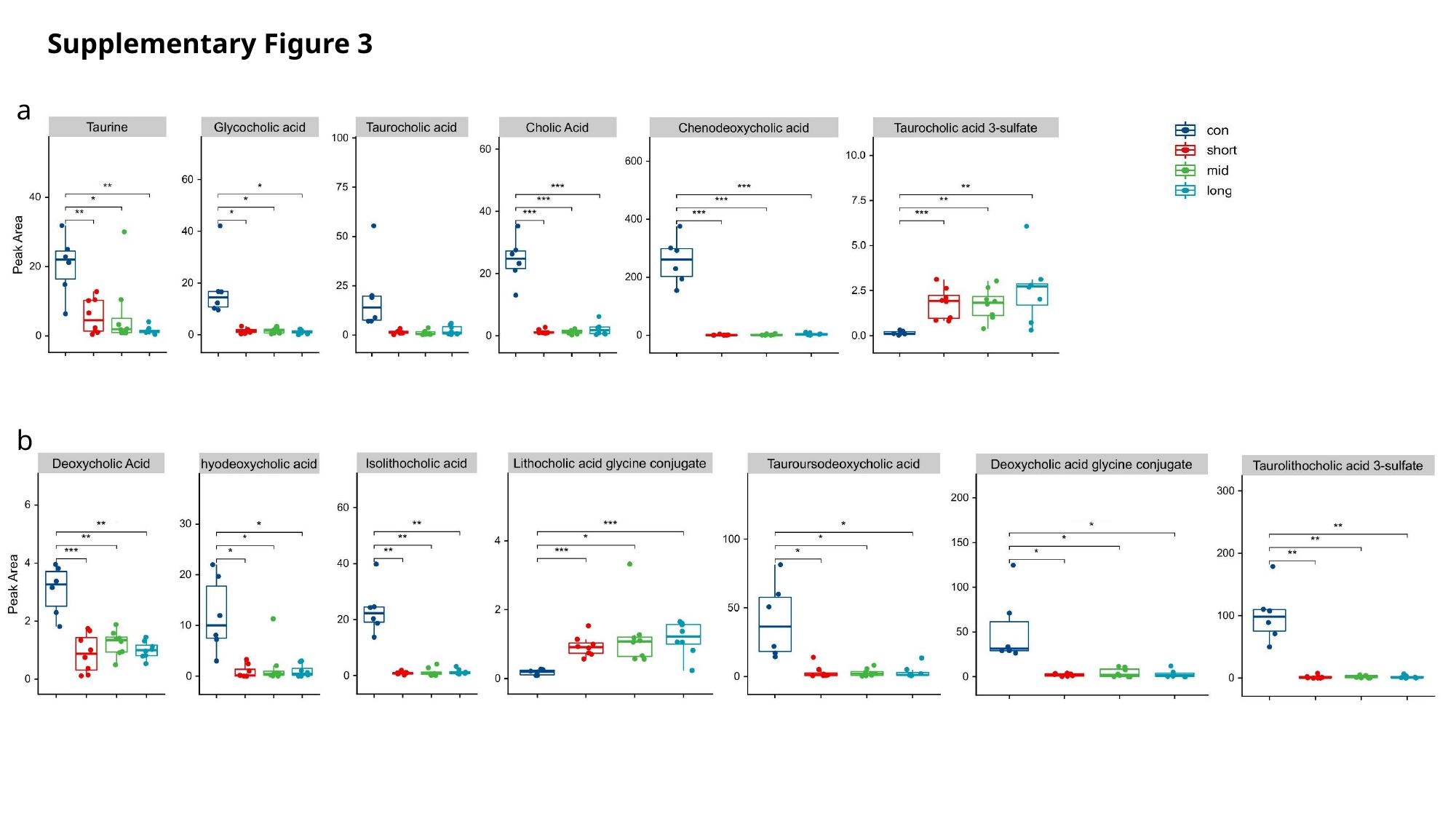

Supplementary Figure 3
a
b

## Slide 4
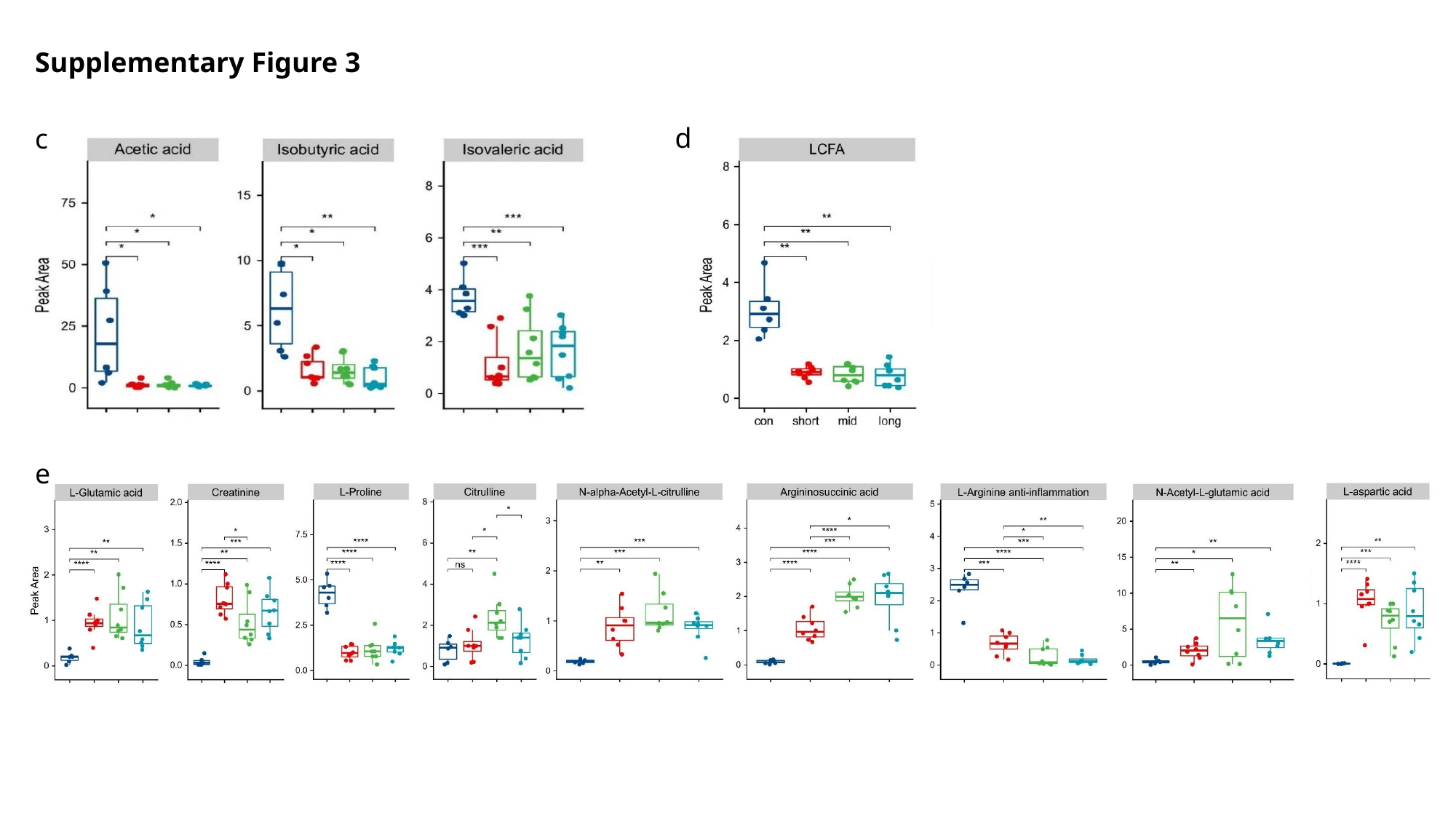

Supplementary Figure 3
d
c
e

## Slide 5
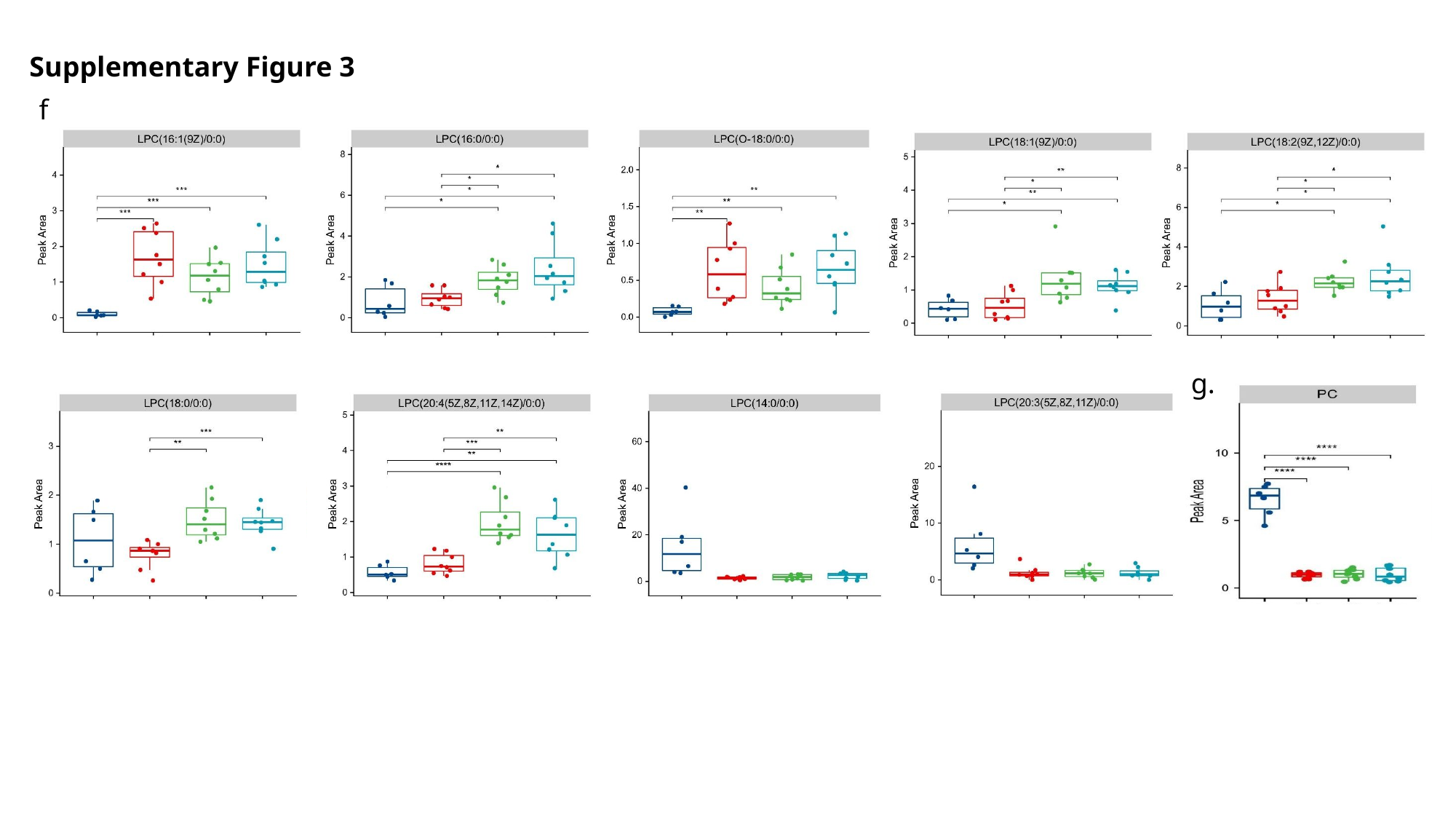

Supplementary Figure 3
f
g.

## Slide 6
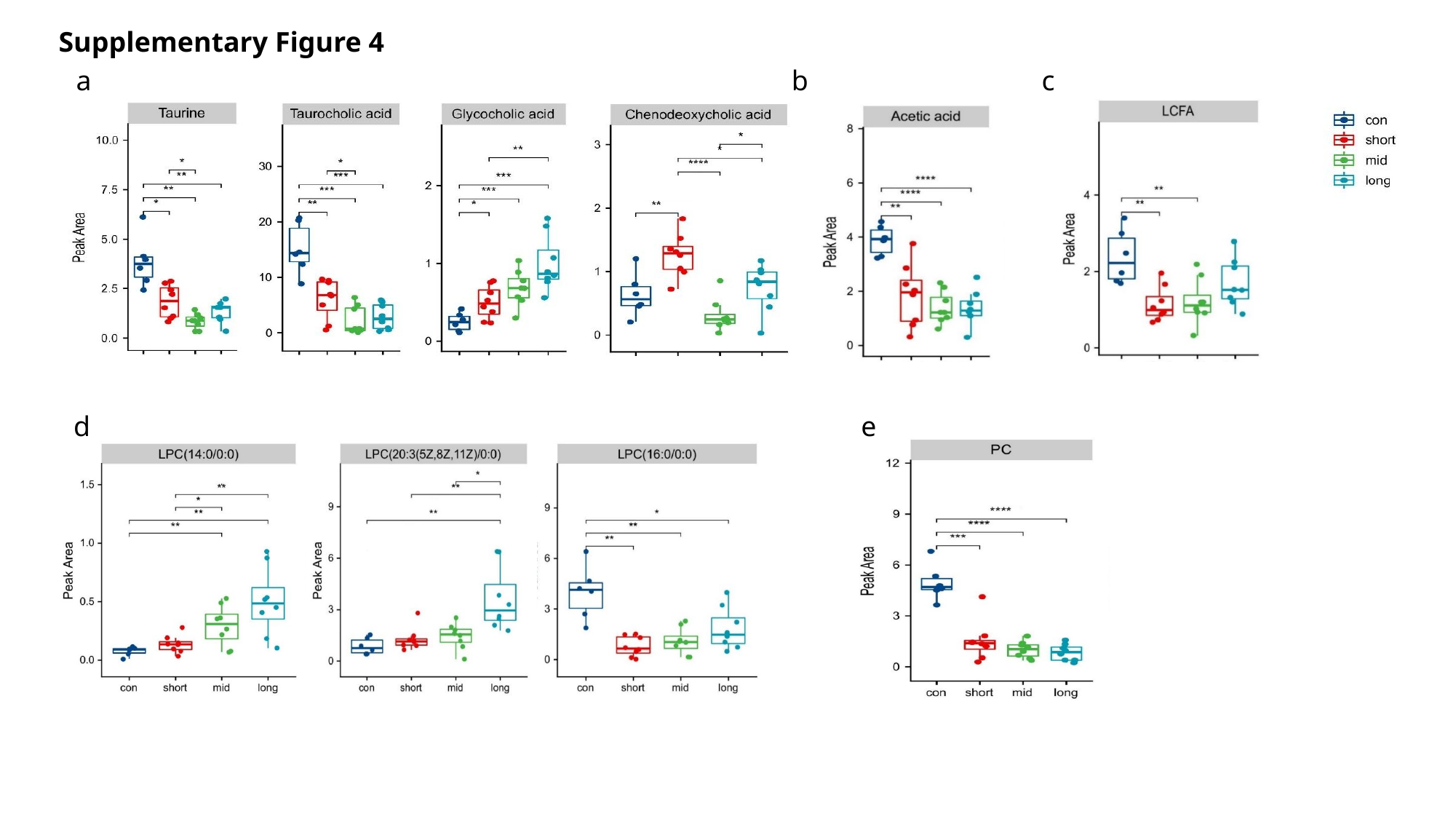

Supplementary Figure 4
a b c
d e
