# Supplementary material for: Integrated Analysis of the Alterations in Gut Microbiota and Metabolites of Mice Induced After Long-Term Intervention With Different Antibiotics
Source: Front Microbiol. 2022 Jun 29;13:832915. doi: 10.3389/fmicb.2022.832915 (PMC9277126; doi:10.3389/fmicb.2022.832915)
Supplement: Supplementary Figure 1 — The histopathology results of colon tissues treated by antibiotics for 8 and 28 weeks (HE stain, X 200). VAN, vancomycin; PMB, polymyxin B; CON, control. [file Data_Sheet_1.zip › Supplementary Tables.DOCX]

**Supplementary Table 1. Vancomycin-induced changes in key metabolites involved in metabolic pathways at different time points.**

| KEGG pathway (ID) | Compound name | Elements | m/z | VAN group1/baseline* | VAN group2/baseline* | VAN group3/baseline * |
| --- | --- | --- | --- | --- | --- | --- |
| Primary bile acids biosynthesis (map00120) | Chenodeoxycholic acid | HMDB0000518 | 375.290 | 0.003 | 0.007 | 0.014 |
|  | Cholic Acid | HMDB0000619 | 426.319 | 0.050 | 0.072 | 0.068 |
|  | Taurocholic acid | HMDB0000036 | 516.270 | 0.083 | 0.072 | 0.095 |
|  | Glycocholic acid | HMDB0000138 | 464.300 | 0.089 | 0.074 | 0.067 |
|  | Taurine | HMDB0000251 | 126.022 | 0.219 | 0.328 | 0.113 |
| Secondary bile acids biosynthesis (map00121) | Taurolithocholic acid 3-sulfate | HMDB0002580 | 562.224 | 0.012 | 0.016 | 0.026 |
|  | Isolithocholic acid | HMDB0000717 | 375.287 | 0.038 | 0.053 | 0.054 |
|  | hyodeoxycholic acid | HMDB0000733 | 783.577 | 0.046 | 0.163 | 0.108 |
|  | Deoxycholic acid glycine conjugate | HMDB0000631 | 448.306 | 0.047 | 0.076 | 0.057 |
|  | Tauroursodeoxycholic acid | HMDB0000874 | 498.289 | 0.079 | 0.072 | 0.076 |
|  | Deoxycholic Acid | HMDB0000626 | 393.300 | 0.257 | 0.406 | 0.358 |
|  | Lithocholic acid glycine conjugate | HMDB0000698 | 434.329 | 4.816 | 6.931 | 6.356 |
| Short-chain fatty acids biosynthesis (map01110) | Acetic acid | HMDB0000042 | 59.017 | 0.057 | 0.057 | 0.041 |
|  | Isobutyric acid | HMDB0001873 | 111.043 | 0.253 | 0.252 | 0.155 |
|  | Isovaleric acid | HMDB0000718 | 103.074 | 0.307 | 0.457 | 0.436 |
| Long-chain fatty acids biosynthesis (map04974) | Dodecanedioic acid | HMDB0000623 | 229.144 | 0.001 | 0.001 | 0.001 |
|  | Behenic acid | HMDB0000944 | 358.331 | 0.002 | 0.001 | 0.001 |
|  | Hexadecanedioic acid | HMDB0000672 | 287.186 | 0.062 | 0.050 | 0.041 |
|  | Arachidonic acid | HMDB0001043 | 303.219 | 0.083 | 0.079 | 0.074 |
|  | 2-Hydroxyhexadecanoic acid | HMDB0031057 | 271.226 | 0.087 | 0.064 | 0.075 |
|  | Heptadecanoic acid | HMDB0002259 | 269.208 | 0.090 | 0.110 | 0.110 |
|  | Prostaglandin E2 | HMDB0001220 | 351.218 | 0.107 | 0.056 | 0.036 |
|  | Stearic acid | HMDB0000827 | 285.276 | 0.135 | 0.079 | 0.112 |
|  | Capric acid | HMDB0000511 | 173.164 | 0.160 | 0.087 | 0.098 |
|  | Palmitoleic acid | HMDB0003229 | 255.231 | 0.256 | 0.171 | 0.181 |
|  | Ricinoleic acid | HMDB0034297 | 297.243 | 0.265 | 0.228 | 0.173 |
|  | Fumaric acid | HMDB0000134 | 114.989 | 0.659 | 0.500 | 0.255 |
| Arginine biosynthesis (map 00220) | L-Arginine | HMDB0000517 | 175.147 | 0.281 | 0.111 | 0.070 |
|  | L-Proline | HMDB0000162 | 231.169 | 0.238 | 0.277 | 0.285 |
|  | Citrulline | HMDB0000904 | 176.104 | 1.358 | 3.025 | 1.624 |
|  | N-alpha-Acetyl-L-citrulline | HMDB0000856 | 240.095 | 4.658 | 6.087 | 4.475 |
|  | Argininosuccinic acid | HMDB0000052 | 308.159 | 12.459 | 23.522 | 22.755 |
|  | Creatinine | HMDB0000562 | 227.121 | 17.298 | 11.198 | 14.054 |
| Tyrosine biosynthesis (map00350) | Tyrosol | HMDB0004284 | 137.061 | 0.002 | 0.002 | 0.002 |
|  | L-Dopamine | HMDB0000181 | 196.046 | 0.027 | 0.050 | 0.034 |
|  | L-Tyrosine | HMDB0000158 | 180.064 | 0.225 | 0.197 | 0.071 |
|  | Normetanephrine | HMDB0000819 | 184.097 | 0.300 | 0.210 | 0.225 |
|  | Gentisic acid | HMDB0000152 | 153.057 | 0.308 | 0.248 | 0.209 |
|  | Dopamine | HMDB0000073 | 154.085 | 0.465 | 0.367 | 0.304 |
|  | Vanillylmandelic acid | HMDB0000291 | 199.057 | 0.507 | 0.495 | 0.272 |
|  | Homovanillic acid | HMDB0000118 | 183.064 | 7.906 | 6.069 | 5.717 |
| Tryptophan biosynthesis (map00380) | Acetyl-N-formyl-5-methoxy kynurenamine | HMDB0004259 | 263.066 | 0.138 | 0.106 | 0.100 |
|  | Indoleacetic acid | HMDB0000197 | 176.070 | 0.170 | 0.198 | 0.099 |
|  | 5-Hydroxy indoleacetic acid | HMDB0000763 | 190.052 | 0.172 | 0.242 | 0.164 |
|  | 4,6-Dihydroxyquinoline | HMDB0004077 | 160.038 | 0.172 | 0.086 | 0.112 |
|  | Kynurenic acid | HMDB0000715 | 379.093 | 0.172 | 0.207 | 0.280 |
|  | Serotonin | HMDB0000259 | 177.112 | 0.191 | 0.424 | 0.206 |
|  | 5-Hydroxykynurenine | HMDB0012819 | 225.091 | 0.212 | 0.295 | 0.429 |
|  | Quinolinic acid | HMDB0000232 | 166.019 | 0.252 | 0.365 | 0.019 |
|  | L-Kynurenine | HMDB0000684 | 209.115 | 2.496 | 2.362 | 1.891 |
|  | 4-(2-Amino-3-hydroxyphenyl)-2,4-dioxobutanoic acid | HMDB0004083 | 222.039 | 2.882 | 3.564 | 55.125 |
|  | Xanthurenic acid | HMDB0000881 | 204.028 | 3.399 | 4.429 | 3.216 |
|  | Indole acetaldehyde | HMDB0001190 | 160.059 | 5.996 | 7.945 | 3.901 |
|  | 4-(2-Aminophenyl)-2,4-dioxobutanoic acid | HMDB0000978 | 206.045 | 6.194 | 11.753 | 7.697 |
|  | 5-Hydroxy-L-tryptophan | HMDB0000472 | 219.077 | 8.178 | 8.001 | 8.150 |
| Glycerophospholipid metabolism (map00564) | LPC(16:1(9Z)/0:0) | HMDB0010383 | 494.324 | 16.716 | 11.266 | 14.683 |
|  | LPC(O-18:0/0:0) | HMDB0011149 | 510.390 | 7.874 | 5.080 | 8.297 |
|  | LPC(20:4(5Z,8Z,11Z,14Z)/0:0) | HMDB0010395 | 544.337 | 1.419 | 3.466 | 2.849 |
|  | LPC(18:2(9Z,12Z)/0:0) | HMDB0010386 | 520.339 | 1.290 | 2.087 | 2.373 |
|  | LPC(16:0/0:0) | HMDB0010382 | 496.339 | 1.228 | 2.335 | 3.103 |
|  | LPC(18:1(9Z)/0:0) | HMDB0002815 | 522.352 | 1.187 | 3.026 | 2.539 |
|  | LPC(18:0/0:0) | HMDB0010384 | 546.353 | 0.727 | 1.384 | 1.329 |
|  | LPC(20:3(5Z,8Z,11Z)/0:0) | HMDB0010393 | 546.350 | 0.193 | 0.186 | 0.196 |
|  | LPC(P-18:1(9Z)/0:0) | HMDB0010408 | 506.356 | 0.147 | 0.120 | 0.215 |
|  | LPC(14:0/0:0) | HMDB0010379 | 468.308 | 0.096 | 0.117 | 0.154 |
| Alanine, aspartate and glutamate metabolism (map00250) | N-Acetyl-L-aspartic acid | HMDB0000812 | 176.055 | 188.766 | 156.840 | 126.195 |
|  | beta-Alanine | HMDB0000056 | 90.054 | 12.121 | 15.719 | 12.491 |
|  | N-Acetylaspartylglutamic acid | HMDB0001067 | 305.076 | 6.698 | 3.550 | 3.601 |
|  | L-Glutamic acid | HMDB0000148 | 146.047 | 5.062 | 5.813 | 4.701 |
|  | N-Acetyl-L-glutamic acid | HMDB0001138 | 188.052 | 4.150 | 12.559 | 7.053 |
|  | Ureidosuccinic acid | HMDB0000828 | 177.052 | 3.085 | 5.094 | 4.319 |
|  | Citric acid | HMDB0000094 | 193.048 | 2.793 | 2.020 | 2.704 |

*all p value < 0.05, group 1, 2 and 3 referred to the timing of treatment for 3-, 5-, and 8-week, for 13- and 18-week and for 23- and 28-week, respectively.

Abbreviations: LPC, lysophosphatidylcholine; HMDB, the Human Metabolome Database; tR, retention time; KEGG, Kyoto Encyclopedia of Genes and Genomes.

**Supplementary Table 2. Polymyxin-induced changes in key metabolites involved in metabolic pathways at different time points**

| KEGG pathway (ID) | Compound name | Elements | m/z | PMB group1/baseline* | PMB group2/baseline* | PMB group3/baseline* |
| --- | --- | --- | --- | --- | --- | --- |
| Primary bile acids biosynthesis (map00120) | Taurine | HMDB0000251 | 125.9866 | 0.479 | 0.348 | 0.219 |
|  | Taurocholic acid | HMDB0000036 | 516.2647 | 0.401 | 0.150 | 0.191 |
|  | Chenodeoxycholic acid | HMDB0000518 | 391.2732 | 1.980 | 0.489 | 1.172 |
|  | Glycocholic acid | HMDB0000138 | 466.3499 | 2.022 | 2.770 | 4.058 |
| Fatty acids biosynthesis (map01110) | Acetic acid | HMDB0000042 | 61.0084 | 0.477 | 0.359 | 0.355 |
| Vitamin B6 metabolism (map00750) | Pyridoxal | HMDB0001545 | 168.0755 | 0.490 | 0.267 | 0.288 |
| Glycerophospholipid metabolism (map00564) | LPC(14:0/0:0) | HMDB0010379 | 466.262 | 1.785 | 3.852 | 6.532 |
|  | LPC(20:3(5Z,8Z,11Z)/0:0) | HMDB0010393 | 546.306 | 1.471 | 1.652 | 4.115 |
|  | LPC(16:0/0:0) | HMDB0010382 | 496.364 | 0.195 | 0.274 | 0.459 |

*all p value < 0.05, group 1, 2 and 3 referred to the timing of treatment for 3-, 5-, and 8-week, for 13- and 18-week and for 23- and 28-week, respectively.

Abbreviations: LPC, lysophosphatidylcholine; HMDB, the Human Metabolome Database; tR, retention time; KEGG, Kyoto Encyclopedia of Genes and Genomes.
